# Diagnostic value of plasma tryptophan and symmetric dimethylarginine levels for acute kidney injury among tacrolimus-treated kidney transplant patients by targeted metabolomics analysis

**DOI:** 10.1038/s41598-018-32958-2

**Published:** 2018-10-02

**Authors:** Feng Zhang, Qinghua Wang, Tianyi Xia, Shangxi Fu, Xia Tao, Yan Wen, Shen’an Chan, Shouhong Gao, Xiaojuan Xiong, Wansheng Chen

**Affiliations:** 1 Department of Pharmacy, Changzheng Hospital, Second Military Medical University, Shanghai, 200003 P. R. China; 2 Key Laboratory of Jiangxi Province for Research on Active Ingredients in Natural Medicines, Bioengineering Research Institute, Yichun University, Yichun, 336000 P. R. China; 3 Department of Pharmacy, Xinqiao Hospital, Third Military Medical University, Chongqing, 400037 P. R. China; 4 Department of Organ Transplantation, Changzheng Hospital, Second Military Medical University, Shanghai, 200003 P. R. China; 5 Agilent Technology, Inc., Taipei, 10492 Taiwan P. R. China

## Abstract

Few literatures have evaluated the exact role of metabolomics in the identification process of potential biomarkers for acute kidney injury among the patients receiving renal transplantation. On top of this, the success of metabolomics in biomarker translation seems to lie in the robust quantitative method. As such, a single-center retrospective observational study was conducted enrolling 42 patients underwent renal transplantation with/without acute kidney injury, as well as 24 healthy volunteers, in Shanghai Changzheng Hospital. Plasma amino acid metabolic patterns for the participants were investigated by targeted UHPLC-MS/MS metabolic profiling. The most significant changes of the explored metabolites were related to the disturbance of tryptophan metabolism and arginine metabolism. Abnormal circulating tryptophan and symmetric dimethylarginine were identified to be potential biomarkers of acute kidney injury, combination of which showed a higher area under receiver-operator curve value (AUC = 0.901), improved sensitivity (0.889) and specificity (0.831) compared with creatinine only. Overall, these results revealed that targeted metabolomics analysis would be a potent and promising strategy for identification and pre-validation of biomarkers of acute kidney injury in renal transplantation patients.

## Introduction

Acute kidney injury (AKI) is increasingly common and associated with transplant failure and death in renal transplantation patients. Its causes are multiple and include rejection, infections, ischemia-reperfusion injury, and drug toxicity and etc^1^. Early detection has proved to be the key to limit the potential damage to transplanted organs and improve long-term prognosis of these receiving kidney transplantation. In addition to kidney biopsy, serum creatinine (SCr) is widely applied as the indicator to monitor the renal function, which is of significant heterogeneity and observed only after substantial kidney injury. While kidney biopsy, an invasive option, could be costly and impractical in multiple sampling. Failure to early diagnosis of AKI often leads to disastrous or even life-threatening episodes of renal dysfunction, thus it is desirable to discover ideal biomarkers, which should be non-invasive and identify AKI in renal transplantation patients.

Metabolomics, an approach for precision medicine, has been yielding important insights into the underlying reasons for acute kidney injury, as well as the biomarker identification. Nevertheless, few metabolomics studies have been conducted in the renal transplantation patients^2^. Of note, the success of metabolomics in biomarker translation seems to lie in the fact that the underlying instrumentation is robust, quantitative, easily adapted to new assays and already located in many clinical testing laboratories^3^, and the clinical merit of potential biomarkers needs to be validated in a well-planned separate targeted study^4^. As a result, the targeted metabolomics approach is reckoned to be preferable for assessment of specific pathways with focus of interest recently, for instance, the newborn screening of inborn errors of metabolism would facilitate doctors to prevent or reduce significant morbidity and mortality in the early stage, by analyzing metabolic patterns of vitamins, amino acids, acylcarnitines, and so on^5–7^.

Plasma free amino acids (PFAAs) played a fundamental role in the metabolism realm as the direct response of metabolic flux, and kidney was shown to play a central role in modulation of AA metabolism. Abnormal plasma AA concentrations had been confirmed to be associated with renal diseases. Once renal dysfunction occurs, both the lost control of AA metabolism by kidney as well as the impact of renal failure and acidosis on whole-body nitrogen metabolism would contribute to the disturbed plasma AA levels^8^. Data from previous researches had shown promising evidence of AA analytics in monitoring the progress of kidney diseases^9^. AA profile in patients’ plasma and urine were of relevance with their clinical evaluation not only in different stages of chronic kidney disease^10^, but also in the AKIs^11,12^. In spite of the established association between plasma AA concentrations with estimated glomerular filtration rate (eGFR) in transplantation patients^13^, little information was available about the value of AA profile in prediction of AKI among patients receiving the renal transplantation. Furthermore, our previous research revealed that concentrations of total AAs and many AAs, such as alanine, lysine, aspartic acid, serine, and methionine, were significantly altered in renal transplantation patients when compared to the healthy volunteers^14^. This finding shed light on the meaning of the AA profile in renal transplantation patients, characterization of which would be necessary for understanding AKI process and identifying potential biomarkers for kidney dysfunction.

Therefore, we herein present a quantitative method of 25 AAs in plasma samples by ultra-high performance liquid chromatography-tandem mass spectrometry (UHPLC–MS/MS)^14^, which was based on modification of our previously published method. Furthermore, the targeted metabolomics study was conducted in 42 patients underwent renal transplantation with/without acute kidney injury and 24 healthy volunteers, in a single-center retrospective observational study. In this pre-validation study, the diagnostic accuracy of potential AA biomarkers was investigated using receiver operating characteristic (ROC) analysis.

## Results

### Clinical Features of participants

Characteristics of the recruited participants were summarized (Table 1), enrolling 24 healthy volunteers (HV group), 12 patients without AKI (NA group) and 30 patients suffering from AKI (NB group). No significant differences of body mass index (BMI) were found among the data sets. Compared with the NA group, patients in the NB group had higher FK506 D_0_/C_0_ (ng/ng/mL), SCr (μmol/L), BUN (mmol/L), UA (μmol/L) and LDH (U/L). However, eGFR (mL/min/1.73 m^2^), HGB (g/L), RBC (10^12/L), LYM (10^9/L), T-BIL (μmol/L) and ALT (U/L) were significantly lower in the NB group. There were no significant differences in other characteristics.

**Table 1:** The characters and biochemical indicators of HV, NA and NB patients.

Clinical features	HV group (n = 24)	NA group (n = 30)	NB group (n = 12)
Gender (man/woman)	24 (13/11)	30 (15/15)	12 (8/4)
Age (year)	24.51 ± 3.16	36.27 ± 8.86	40.25 ± 14.40
FK506 C_0_ (ng/mL)	—	10.98 ± 1.63	11.77 ± 5.57
FK506 D_0_/C_0_ (ng/ng/mL)	—	208.37 ± 69.51	268.05 ± 76.71*
eGFR (mL/min/1.73 m^2^)	99.42 ± 11.61	108.4 ± 19.34	35.92 ± 18.94*
SCr (μmol/L)	83.04 ± 14.82	83.7 ± 15.01	250.58 ± 88.76*
BUN (mmol/L)	4.47 ± 1.23	5.49 ± 0.96	17.18 ± 5.82*
UA (μmol/L)	295.75 ± 78.94	308.50 ± 68.26	476.27 ± 93.47*
GLU (mmol/L)	4.82 ± 0.28	4.88 ± 0.74	4.88 ± 0.80
TP (g/L)	73.98 ± 3.65	66.64 ± 5.74	64.80 ± 7.93
ALB (g/L)	48.22 ± 2.13	42.82 ± 4.91	41.90 ± 6.15
GLB (g/L)	25.76 ± 2.68	23.82 ± 4.85	23.20 ± 3.80
A/G (g/g)	1.89 ± 0.21	1.88 ± 0.45	1.84 ± 0.33
WBC (10^^9^/L)	6.03 ± 1.53	7.50 ± 2.90	7.05 ± 3.65
HGB (g/L)	139.04 ± 10.88	105.96 ± 20.19	91.20 ± 9.46*
RBC (10^^12^/L)	5.09 ± 0.45	3.52 ± 0.65	2.97 ± 0.36*
LYM (10^^9^/L)	2.39 ± 0.78	1.73 ± 0.84	1.00 ± 0.49*
PLT (10^^9^/L)	224.12 ± 38.58	229.26 ± 73.16	258.30 ± 86.66
T-BIL (μmol/L)	9.69 ± 2.51	9.96 ± 2.18	8.00 ± 2.54*
AST (U/L)	22.52 ± 5.04	24.52 ± 19.27	20.70 ± 6.15
ALT (U/L)	15.75 ± 6.52	37.38 ± 45.52	15.00 ± 4.97*
LDH (U/L)	202.57 ± 45.01	169.92 ± 30.26	249.86 ± 83.98*
U-WBC (/μL)	16.42 ± 6.07	18.56 ± 26.05	8.29 ± 4.44
U-RBC (/μL)	17.53 ± 5.49	21.75 ± 58.28	5.69 ± 5.14
Body weight (kg)	62.57 ± 11.96	55.33 ± 12.50	54.63 ± 12.86
Clinical history (renal dysplasia, year)	—	10.06 ± 7.80	8.75 ± 5.85

*NB group VS. HV group in Tukey test.

Data was presented as mean ± standard deviation.

Renal function indexes examined included serum creatinine (SCr), blood urea nitrogen (BUN), estimated glomerular filtration rate (eGFR) and uric acid (UA). Blood routine indexes examined included total protein (TP), albumin (ALB), globulin (GLOB), albumin/globulin (A/G), white blood cell (WBC), hemoglobin (HGB), red blood cell (RBC), lymphocyte (LYM), blood platelet (PLT) and glucose (GLU). Liver function indexes examined included total bilirubin (TBIL), alanine aminotransferase (ALT), aspartate transaminase (AST), lactate dehydrogenase (LDH). Urine routine indexes examined included urine white blood cell count (U-WBC), urine red blood cell count (U-RBC), pH and specific gravity. FK506 dose (D_0_), FK506 trough concentration (FK506 C_0_), and their ration FK506 D_0_/C_0_ for each kidney transplantation patient were recorded.

### Validation of the UHPLC-MS/MS analytical method for AAs

Mixed solutions of 25 AAs were employed for method validation (SI, Text S1). L-alanine-d4 (L-Ala-d4), L-methionine-d3 (L-Met-d3) and L-phenylalanine-d5 (L-Phe-d5) were applied as internal standards (SI, Text S2). Preparation for the standards and quality control (QC) samples was described in SI, Text S2. The developed UHPLC-MS/MS method was validated for linearity, precision, accuracy, matrix effect, recovery, incurred sample reanalysis (ISR) and stability, according to recommendations published by FDA (US Food and Drug Administration, 2013)^15^ (SI, Text S3). Linear equations were obtained with acceptable linear correlation coefficients (r ≥ 0.99) (SI, Table S1). Three different levels of QC were employed for the inter- and intra-day precision analysis, with coefficient of variation (CV%) ranged from 3.0% to 4.1%, and from 1.7% to 2.8%, respectively. Accuracy for the above samples was expressed as relative error (RE%), which was in the range of −14.52% to 14.92% (SI, Table S2). Matrix effect and recovery results turned out to be stable and repeatable for all the analytes, with the values of 89.96% (range 80.01−107.19%, except Oxo) and mean recovery was 94.02% (range 81.01–110.38%), respectively (SI, Table S3). RE for the ISR was calculated within 20%. QC samples were found to be stable (RE ≤ 20%) in the stability tests, including short-term stability, post-preparation stability, three freeze-thaw cycle stability and long-term stability (SI, Table S4).

### Metabolic profiling of healthy volunteers and kidney transplantation patients

**Figure 1:**
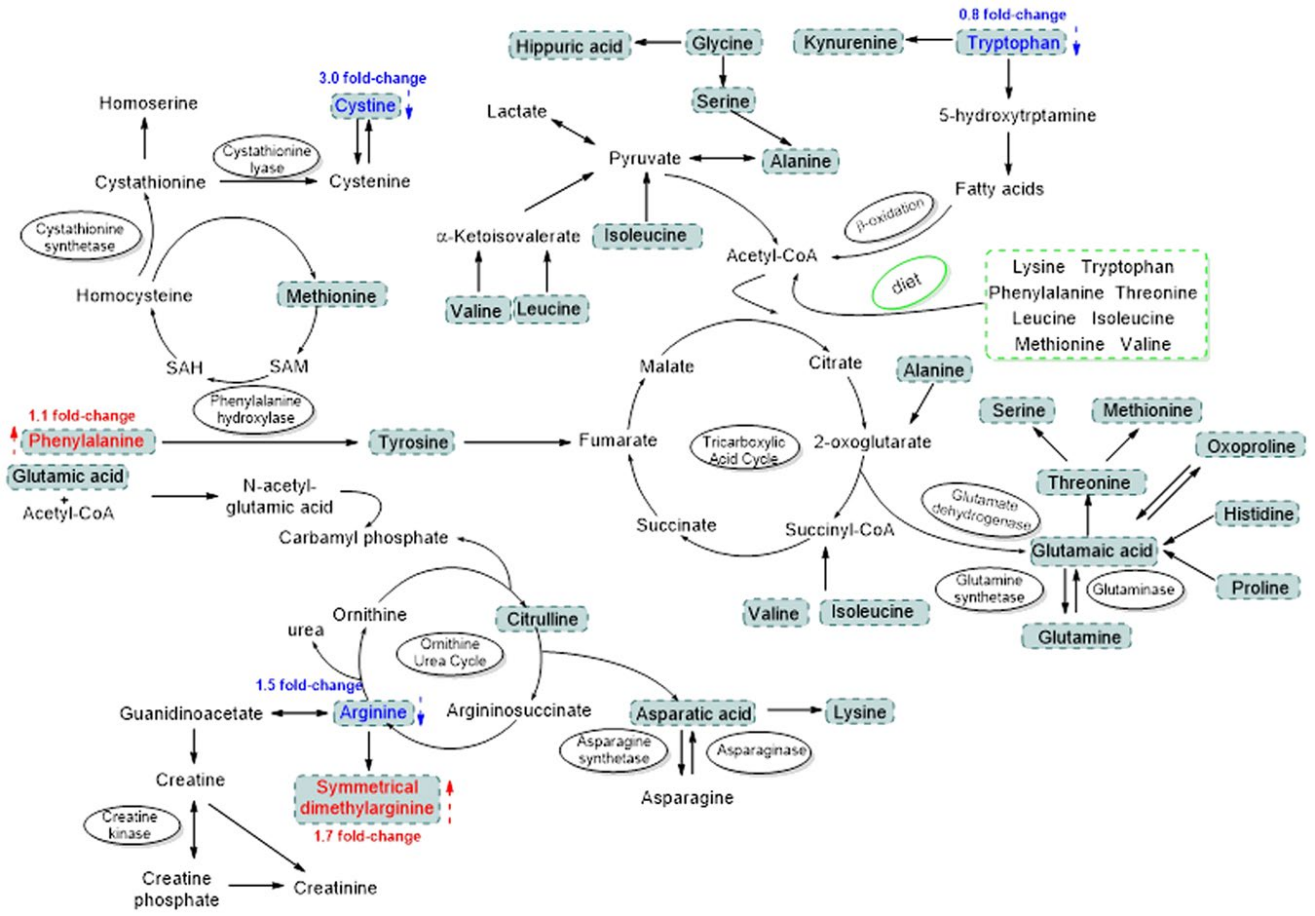
Investigated AA metabolic pathways and the potential biomarkers with their fold-changes in NA and NB groups. Red arrow and blue arrow means the AA change in the NB group compared with the NA group.

Figure 1 interpreted the investigated AA metabolic pathways, as well as the fold-changes of the potential biomarkers in NB group when compared with those in NA group^16^. Effective quantification of plasma AAs was achieved using the validated method in plasma samples from 24 healthy volunteers and 42 patients. Multi-group comparisons through ANOVA analysis (p < 0.05) indicated a different metabolic pattern for most studied AA. To be specific, NA and NB patients had a lower level of AA profile, such as Cys, HA, Arg, His, Trp, Gln/Glug, Arg/Phe, Ser/Gly, Val/Gly, compared with those from HV subjects (p < 0.05) (Table 2). Furthermore, NB patients had much lower concentrations of Trp and ratios of in Leu/Ile, Pro/Cit and SDMA/SCr, but higher levels of SDMA, Phe and Kyn/Trp, compared with those from NA patients (p < 0.05). In detail, AKI patients showed increased levels of SDMA, Phe and Kyn/Trp ratio, with values of 0.2547~0.4962 μg/mL, 10.7692~23.9799 μg/mL and 0.0364~0.1093, respectively, but decreased levels of Trp and SDMA/SCr ratio, with values of 6.7584~13.8741 μg/mL and 0.9510~3.1014, respectively. Among the above mentioned, the levels of Ser and Lys in our study were lower than those reported in other studies^17,18^, which might be derived from different AKI status of patients or others.

**Table 2:** Quantification result of 25 amino acids and related ratios in three individuals.

Parameters	HV group (n = 24)	NA group (n = 30)	NB group (n = 12)
Gly	8.55 ± 0.98	12.42 ± 5.43^a^	12.93 ± 4.50^b^
Ala	36.22 ± 7.70	48.28 ± 19.18^a^	50.30 ± 20.41^b^
Ser	3.20 ± 0.49	3.09 ± 1.46	3.60 ± 1.61
Pro	35.42 ± 11.90	32.08 ± 23.66	38.36 ± 24.40
Val	21.48 ± 3.78	21.22 ± 2.95	21.05 ± 3.61
Thr	5.00 ± 1.03	7.65 ± 3.51^a^	8.11 ± 4.78^b^
Oxo	9.34 ± 1.28	21.27 ± 11.83^a^	18.02 ± 8.30^b^
Leu	16.24 ± 3.08	18.06 ± 3.18	18.12 ± 3.07
Iso	9.05 ± 1.79	9.57 ± 2.07	10.73 ± 2.29
Asp	1.66 ± 0.16	1.24 ± 0.94	0.76 ± 0.34^b^
Gln	11.18 ± 1.36	9.16 ± 5.96^a^	12.43 ± 5.71^b^
Lys	0.14 ± 0.06	0.22 ± 0.07^a^	0.37 ± 0.08^b,c^
Glu	1.14 ± 0.34	6.72 ± 4.22^a^	6.65 ± 5.40^b^
Met	4.75 ± 0.81	3.64 ± 0.85^a^	4.52 ± 2.22
His	3.45 ± 0.51	1.85 ± 0.55^a^	2.03 ± 0.57^b^
Phe	11.42 ± 1.32	13.54 ± 2.33^a^	14.80 ± 3.77^b,c^
Arg	2.88 ± 0.97	1.11 ± 0.94^a^	1.65± 0.79^b,c^
Cit	10.17 ± 1.68	6.85 ± 7.97^a^	12.39 ± 8.30^c^
HA	0.58 ± 0.75	0.15 ± 0.16^a^	0.17 ± 0.17^b,c^
Tyr	10.60 ± 1.27	9.60 ± 2.11	9.65 ± 2.70
SDMA	0.10 ± 0.02	0.22 ± 0.07^a^	0.37 ± 0.08^b,c^
Trp	14.10 ± 2.59	12.28 ± 2.36^a^	9.94 ± 2.30^b,c^
Kyn	0.42 ± 0.09	0.50 ± 0.27	0.59 ± 0.22^a^
Cys	0.64 ± 0.18	0.10 ± 0.10^a^	0.30 ± 0.28^b,c^
Leu/Iso	1.80 ± 0.14	1.92 ± 0.22	1.71 ± 0.23^c^
Gln/Glu	10.39 ± 2.65	1.89 ± 1.19^a^	2.77 ± 2.12^b^
ALB/Leu	3.04 ± 0.55	2.42 ± 0.41^a^	2.38 ± 0.34^b^
ALB/Iso	5.48 ± 1.14	4.68 ± 1.23^a^	4.07 ± 0.40^b^
ALB/Val	2.28 ± 0.38	2.04 ± 0.33	2.09 ± 0.34
Pro/Cit	3.57 ± 1.29	14.14 ± 17.20	3.27 ± 1.74^c^
Phe/Tyr	1.08 ± 0.10	1.48 ± 0.40^a^	1.62 ± 0.50^b^
Kyn/Trp	0.03 ± 0.01	0.04 ± 0.03	0.06 ± 0.02^c^
Arg/Phe	0.25 ± 0.09	0.08 ± 0.08^a^	0.12 ± 0.07^b,c^
SDMA/SCr	1.13 ± 0.18	2.65 ± 0.88^a^	1.65 ± 0.61^b,c^
Ser/Gly	0.37 ± 0.04	0.27 ± 0.13^a^	0.27 ± 0.07 ^b^
Val/Gly	2.57 ± 0.66	2.01 ± 0.83	1.85 ± 0.80^b^
Arg/Cit	0.29 ± 0.10	0.37 ± 0.44	0.24 ± 0.25
TAA	222.81 ± 30.08	247.66 ± 61.84	263.84 ± 64.50
TEAA	87.22 ± 13.00	93.00 ± 10.04	95.62 ± 16.55
TNEAA	135.58 ± 18.41	154.66 ± 60.72	168.22 ± 58.37
TBCAA	46.77 ± 8.52	48.86 ± 7.24	49.91 ± 8.20
TAAA	36.11 ± 4.87	35.42 ± 4.48	34.38 ± 6.78
TGAA	144.00 ± 22.07	158.06 ± 44.74	171.15 ± 53.09
TKAA	71.60 ± 9.81	77.73 ± 9.83	79.70 ± 12.74
TGKAA	50.17 ± 6.64	52.65 ± 6.44	53.23 ± 8.24
TBCAA/Tyr	4.40 ± 0.50	5.37 ± 1.54^a^	5.54 ± 1.59^b^
ALB/TBAA	0.78 ± 0.13	1.04 ± 0.59	1.02 ± 0.45
TEAA/TNEAA	0.64 ± 0.05	0.66 ± 0.19	0.63 ± 0.22

^a^NA group VS. HV group, p < 0.05; ^b^NB group VS. HV group, p < 0.05; ^c^NB group VS. NA group, p < 0.05 in Tukey test.

Data was presented as mean ± standard deviation.

Gly, glycine; Ala, alanine; Ser, serine; Pro, Proline; Val, valine; Thr, threonine; Oxo, oxoproline; Leu, leucine; Iso, isoleucine; Asp, aspartic acid; Gln, glutamine; Lys, lysine; Glu, glutamic acid; Met, methionine; His, histidine; Phe, phenylalanine; Arg, argnine; Cit, citrulline; HA, hippuric acid; Tyr, tyrosine; SDMA, symmetric dimethylarginine; Trp, tryptophan; Kyn, kynurenine; Cys, cystine; TAA, total amino acid; TEAA, total essential amino acid; TNEAA, total nonessential amino acid; TBCAA, total branched chain amino acid; TAAA, total aromatic amino acid; TGAA, total glucogenic amino acid; TKAA, total ketogenic amino acid; TGKAA, total glucgenic and ketogenic amino acid.

AMA (aminomalonic acid) concentrations from subjects were below LLOD. AA concentrations were presented in μg/mL.

### Correlation between clinical features and AA profile

**Figure 2:**
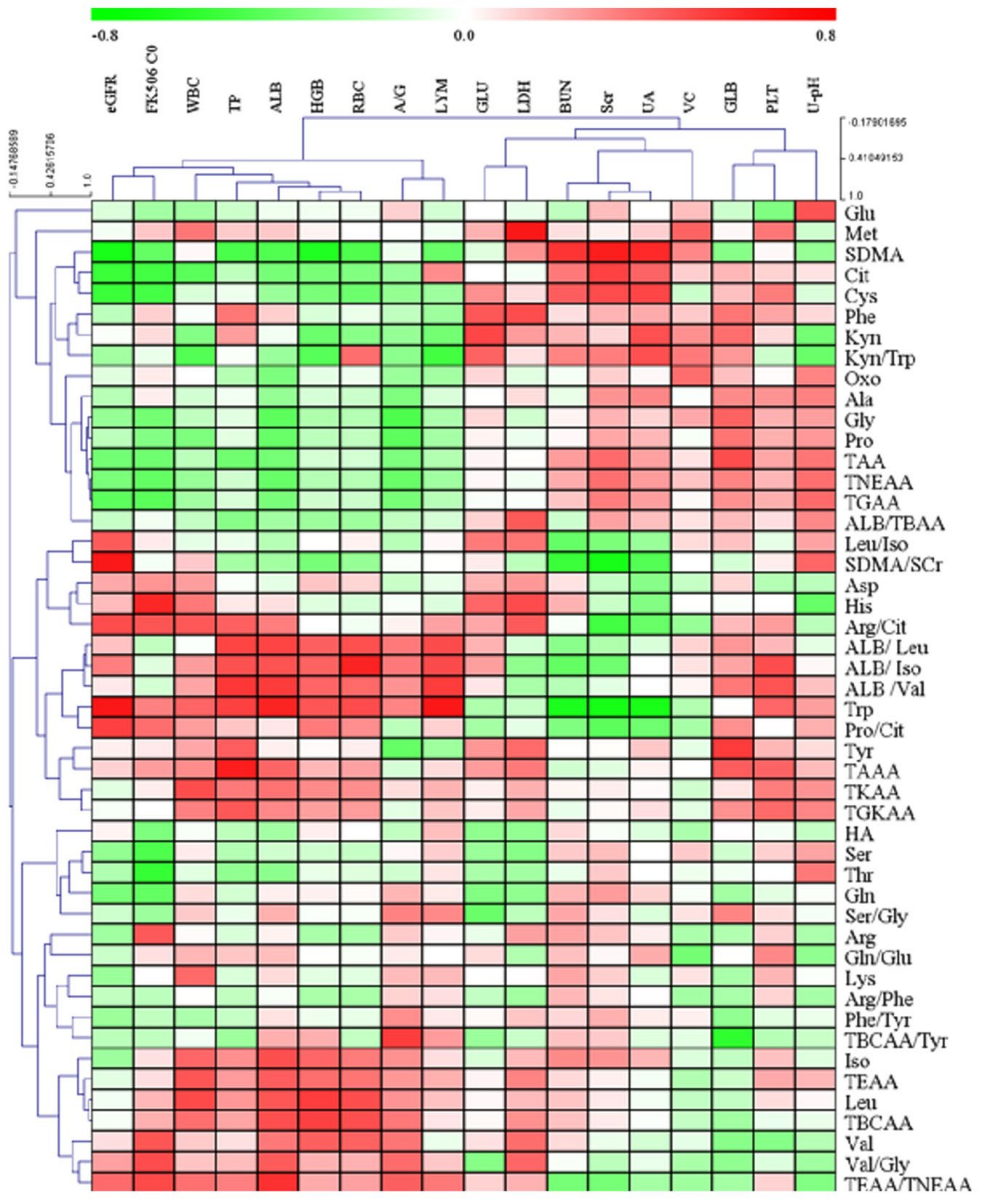
Heat-map and clustering analysis of amino acid concentrations and related ratios with clinical biochemical indexes in NB group. Variable in each row represents an amino acid or a ratio, and each column represents a clinical feature. The correlation coefficients were represented with red for positive correlation and green for negative ones as illustrated in the color key. Variables that showed similar correlation coefficients were clustered together.

Correlation between clinical features and AAs as well as their ratios in NB group was analyzed using Pearson (r) or Spearman (ρ) Rank test, and displayed in a heat-map (Fig. 2). Detailed data were listed in Tables S5a and S5b (SI). The input data was then evaluated by hierarchical clustering analysis, allowing the visualization of the clinical data across multiple subjects with many analytes^19^. Of note, SDMA, Trp and SDMA/SCr showed the most significant negative correlations with BUN, SCr and UA, and the most positive correlations with eGFR. Considering that SCr level was widely applied to assess eGFR for kidney function diagnosis and used as an index of kidney function in clinical, the correlation analysis showed that levels of SDMA, Trp and SDMA/SCr ratio had significant association with kidney function, which was shown in Fig. S1, Tables S6a and S6b (SI) for HV group, and in Fig. S2, Tables S7a and S7b (SI) for NA group.

### Prediction performance by the area under receiver operating characteristic (AUROC) curve analysis

**Figure 3:**
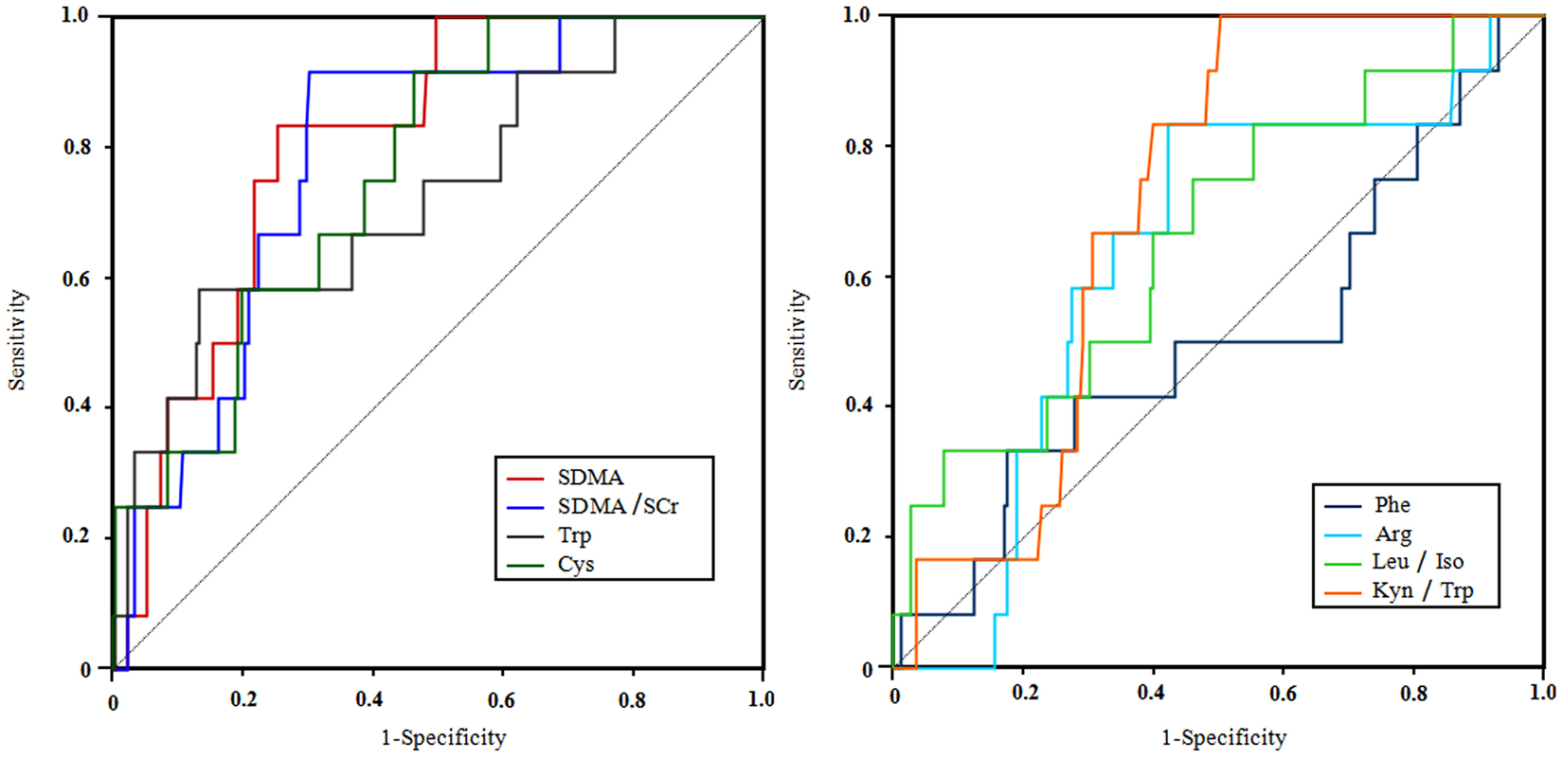
The ROC results of SDMA, SDMA/SCr, Trp, Cys, Phe, Arg, Leu/Iso and Kyn/Trp.

ROC curves were applied to evaluate the diagnostic effectiveness of both potential AA biomarkers, and to find an optimal cut-off value based on Youden index. Therefore, the AUC of ROC curve was computed in the prediction mode. Five indexes, including SDMA, SDMA/SCr, Trp, Cys and Kyn/Trp, were employed as diagnostic biomarkers, which showed AUC values greater than 0.7 in ROC analysis^20,21^ (Fig. 3). When comparisons were made between patients with and without AKI, the AUCs were calculated as 0.820 (95% CI, 0.732 to 0.908; p = 0.000), 0.738 (95% CI, 0.588 to 0.889; p = 0.005), 0.724 (95% CI, 0.608 to 0.840; p = 0.009), 0.785 (95% CI, 0.679 to 0.891; p = 0.001), and 0.709 (95% CI, 0.616 to 0.802; p = 0.015), for levels of SDMA, Trp, Cys, and SDMA/SCr and Kyn/Trp ratios, respectively (SI, Table S8). The AUCs for assessing AKI diagnostic accuracy were found to be 0.820 and 0.738 for SDMA and Trp, respectively, which was the highest among all markers. Moreover, a larger AUC (0.901) was obtained by combination of SDMA and Trp, with a sensitivity of 0.889 and specificity of 0.831 for the diagnosis of AKI (SI, Fig. S3).

## Discussion

Early diagnosis of kidney injury could improve the appropriate drug regime control, resulting in decreased development costs and promising long-term survival rate of such patients. In this single-center retrospective study, we established and validated a UHPLC–MS/MS method for 25 plasma AA determination among three independent groups.

**Figure 4:**
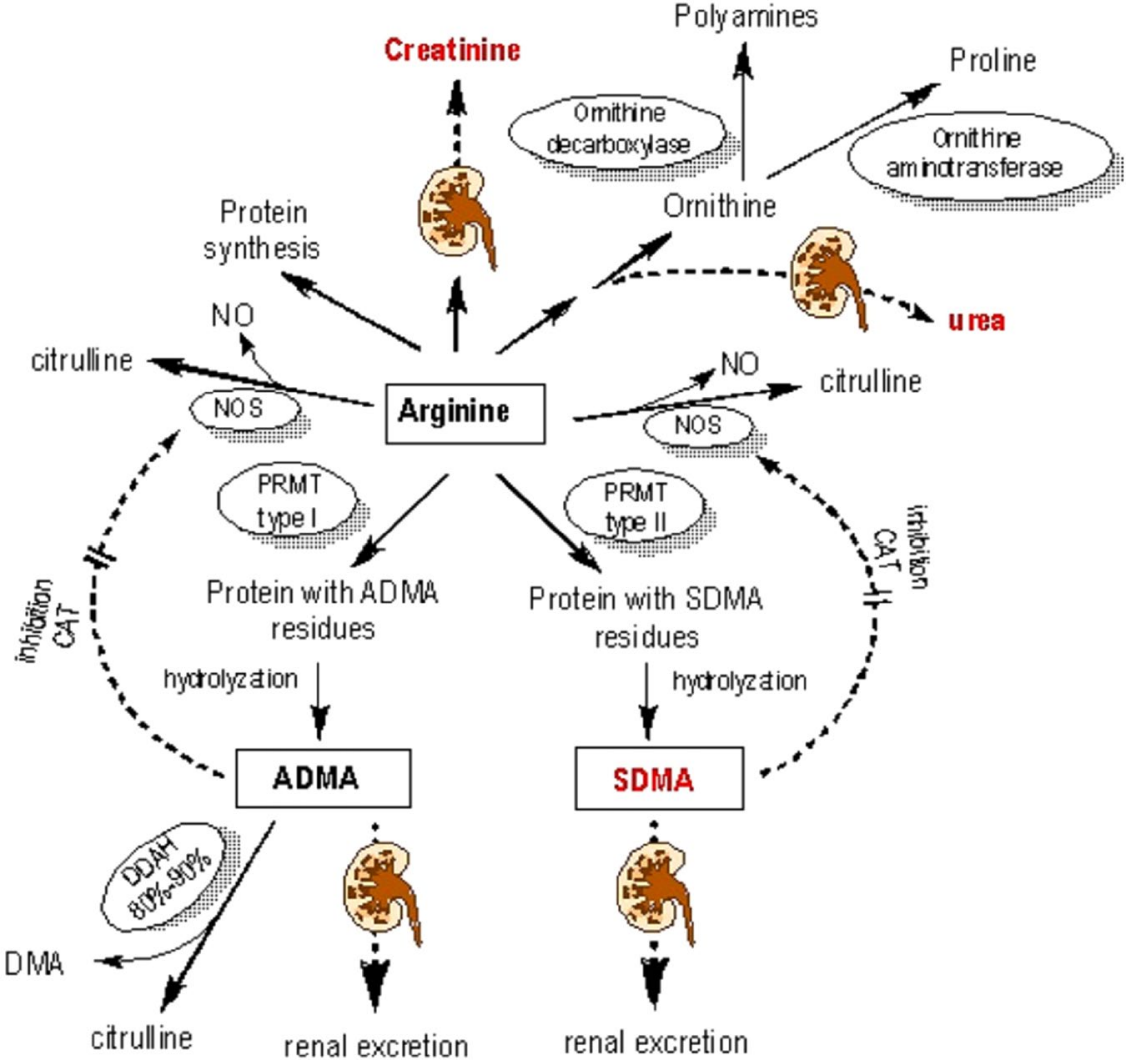
SDMA metabolic pathways. PRMT, protein arginine methyltransferase; NOS, nitric oxide synthase; DDAH, dimethylarginine dimethyl amino hydrolase; DMA, dimethylarginine; CAT, cationic amino acid transporter.

AA profile analysis revealed the perturbed Arg cycle in both NA and NB groups. Arg was degraded to Cit by nitric oxide synthase (NOS), but Arg could be synthesized from Cit via the urea cycle enzymes argininosuccinate synthetase (ASS) and argininosuccinatelyase (ASL), which were mainly located in the proximal tubular cells^22^. In this study, Cit/Arg ratio showed no significant difference between NA and NB groups, although Cit and Arg levels were higher in NB than those in NA group, which might be derived from the potential bidirectional conversion. Similar trends of Cit and Arg level change could also be found in many pathological conditions^23^. In NB group, Cit level increased (about 180%, 1.8 fold-change) compared with NA group, supported its potential role as an early marker for kidney injury, which was in agreement with a previous report^24^. Although both SDMA and ADMA could affect the endogenous deficiency of NO, ADMA was often recognized as an indicator of cardiovascular events and death in different populations. In contrast to ADMA, SDMA was barely catabolized, but excreted exclusively by the kidney, implying that its accumulation could only occur due to the decreased excretion in patients with renal diseases. SDMA was therefore identified as a promising marker of renal function^25^ (Fig. 4). In NB group, levels of Arg and SDMA increased significantly to about 150% (1.5 fold-change) and 170% (1.7 fold-change) in comparison with those of NA group. This was in consistent with the report of a renal ischemia/reperfusion (I/R) in rat model, which indicated that SDMA might contribute to the dysfunction of endothelial cells in such ischemic AKI model^26^. In our experiment, SDMA showed a significant increase in the kidney transplantation patients when compared with that of healthy subjects (0.10 ± 0.02 μg/mL). Furthermore, its plasma level was elevated in patients with AKI (0.37 ± 0.08 μg/mL) in comparison to those without (0.22 ± 0.07 μg/mL). These findings were in accordance with the results derived from much larger populations^27,28^. Convincingly, both SDMA level and SDMA/SCr ratio showed strong correlations with plasma levels of SCr, eGFR, BUN and UA. However, SDMA level showed little correlation with levels of SCr, eGFR, BUN and UA in both HV and NB groups. Similar correlation coefficients were also uncovered by many reports, including meta-analysis and metabolomics studies^29–32^. Additionally, SDMA was reported to be independently associated with graft loss and mortality after adjusting for eGFR in human as well as in rodents^26,33^. To this end, our data firstly presented the promising value of SDMA or SDMA/SCr for prediction of AKI in patients receiving renal transplantation, and revealed that plasma SDMA levels increased in parallel with the rise of SCr, BUN and UA when such patients developed renal injury.

**Figure 5:**
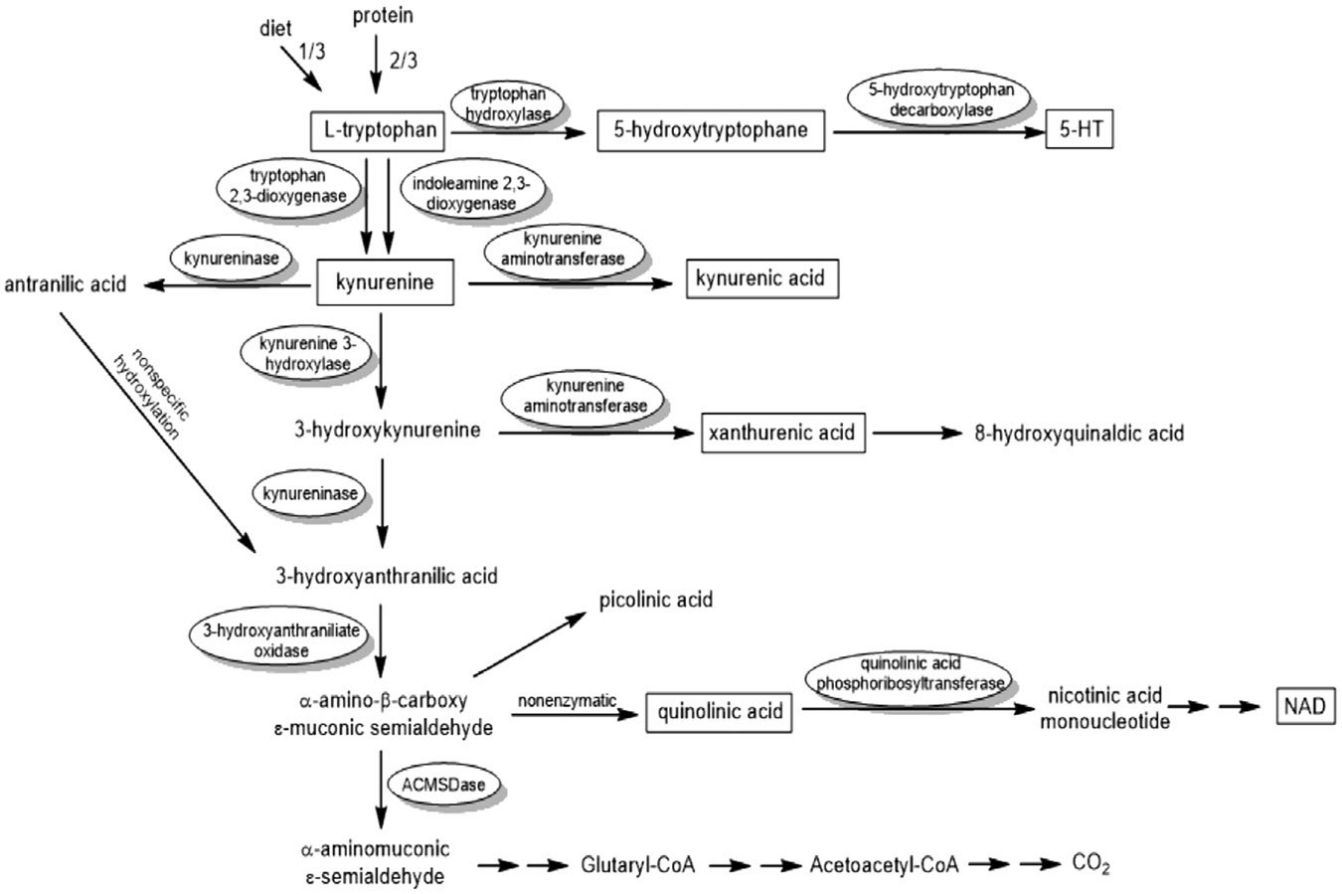
Tryptophan metabolic pathways.

Trp metabolism was highlighted in recent metabolomics researches in patients with kidney cancer^34^. In our study, NB group was elucidated with a marked decrease of Trp (P < 0.01) and slight rise of Kyn, indicating a disturbed Trp metabolism. Enzyme indoleamine 2,3 dioxygenase 1 (IDO1) was the key and rate-limiting enzyme of Trp metabolism, therefore plasma Kyn/Trp ratio was applied as an indicator of the enzymatic function of IDO1 (Fig. 5)^35,36^. In rat and human, renal insufficiency would lead to Trp level reduction and Kyn level accumulation in blood^37^, and the activity of IDO and serum Kyn level increased with the severity of chronic kidney disease (CKD)^38–40^. Likewise, our experiment demonstrated that Trp level significantly decreased from 12.28 ± 2.36 μg/mL in NA group to 9.94 ± 2.30 μg/mL in NB group (0.8 fold-change), accompanied by a rise trend of Kyn level, from 0.50 ± 0.27 μg/mL to 0.59 ± 0.22 μg/mL. In parallel of this, Kyn/Trp ratio was significantly increased from 0.04 ± 0.03 to 0.06 ± 0.02 during episodes of kidney injury.

Some limitations of this analysis demanded further discussion. Firstly, the observed perturbed AA biomarkers could not be substantiated to be associated with the donor and recipient pharmacogenetic factors for AKI in current study. In view of this, further interpretation of the metabolomics, with pharmacogenetic and demographic characters, was still needed for a robust integrated biomarker system. Secondly, only some preselected AAs had been investigated and tested in our study. Because it was becoming increasingly clear that the signs of AA profile disorders would entail and/or accompany with a suite of altered metabolites, which could be analyzed in the future LC-MS/MS based targeted metabolomics method that covered more predefined metabolites. Besides, our results reinforced the concept that AA metabolism were important for the AKI episode. Thirdly, the diagnostic value of the potential AAs or the AA profile should be validated with larger sample volume in clinical practice, and whether the alteration in the plasma AA preceded changes in the classically used biomarkers or other established ones needs to be confirmed. Importantly, future researches should attempt to differentiate the causes of AKI, which was not identified in this study, because it was our preliminary research to correlate the diagnostic performance of targeted AA metabolic profiling analysis with kidney injury.

In summary, we investigated the perturbed AA metabolic pattern in plasma from renal transplantation patients by using targeted metabolomics study, based on a validated UHPLC-MS/MS method. This strategy resulted in the discovery of Arg and Trp cycle disturbances in AKI, with SDMA and Trp as potential risk predictors. Furthermore, SDMA and Trp combination revealed a larger AUC over SCr and any other single AA biomarker. To our knowledge, this was the first study to report that SDMA and/or Trp may function as potential biomarkers for prediction of AKI in renal transplantation patients.

## Methods

### Study population

A retrospective review of a prospectively collected database of kidney transplants at Shanghai Changzheng Hospital from Nov. 2013 to Aug. 2014 revealed 42 hospitalized kidney transplantation patients suitable for the study. Each patient enrolled in this study received a kidney transplantation from a living donor at our institution (Organ Transplantation Center, Changzheng Hospital, Second Military Medical University, Shanghai). Recipients of a deceased donor kidney were not included in this study and no organs were procured from (executed) prisoners. Patients who suffered from diabetes, hypoglycemia, inherited metabolic diseases, and other metabolic disorders were excluded. All the clinical and research activities reported here were in accordance with the Declaration of Helsinki as well as other relevant guidelines and regulations, and approved by the ethical board of Shanghai Changzheng Hospital. Informed consent was retrieved from all the participants. They were treated with tacrolimus (FK506, 0.05 mg/kg), mycophenolate mofetil (1.0 g/d). Corticosteroids were given intravenously during the operation and over the next 2 days. From day 3 post-transplantation, prednisone (0.15 mg/kg) was prescribed with a dose reduced to 10 mg/d at the end of 4 months. Besides, patients were forbidden from any drug or food that could influence the FK506 concentrations. Blood samples for the determination of trough FK506 concentrations (C0) were taken immediately prior to the morning dose for therapeutic drug monitoring (TDM), with C0 maintained at 10–12 ng/mL within the first three post-operative months, 8–10 ng/mL in the post-operative 3–6 months, 6–8 ng/mL in the post-operative 6–12 months, and 4–6 ng/mL thereafter. As a retrospective study, the plasma from the enrolled patients were from the sample after TDM.

Based on KDIGO (Kidney Disease: Improving Global Outcomes) criteria^41,42^, 12 patients were diagnosed with AKI, as there was an increase in SCr of more than 0.3 mg/dL (26.5 μmol/L) or at least a 50% increase of SCr over baseline values. As at least three independent groups were suggested for metabolite biomarkers identification study, healthy control (HV), disease group (NB), and an additional related-disease control group (NA) were investigated in our targeted metabolomics study^4,43^.

All patients were enrolled following the inclusion criteria: subjects were followed up in Shanghai Changzheng Hospital and were clinically stable at the time of the study. Subjects were excluded when any of the following exclusion criteria was met: age under 18 or above 65, being a recipient of a multi-organ transplant, cancer patients, residing abroad, being pregnant, presence of obstructive uropathy which was ruled out by performing a renal ultrasound, and inability to give informed consent. Demographics and clinical data of subjects investigated were listed in Table S1. eGFRs were calculated with the abbreviated Modification of Diet in Renal Disease study (MDRD) equations^44^.

### Sample collection and preparation

Blood samples were taken in the morning after an overnight fasting in EDTA-3K tubes, and centrifuged at 4000 × *g* for 10 min at 4 °C. Plasma samples were stored at −80 °C before analysis.

Thawed plasma sample (100 μl) was treated with acetonitrile (300 μl, containing 0.2% formic acid and 400 ng/ml ISs) in 1.5 ml Eppendorf tubes for sample preparation. The mixture was vortexed and then centrifuged at 12,000 × *g* for 15 min at 4 °C. The supernatant was transferred to chromatography vials, prior to UHPLC–MS/MS analysis.

### Metabolic profiling analysis

Targeted AA metabolic profiling was obtained by a non-derivatization UHPLC–MS/MS method, applying heptafluorobutyric acid as ion-pair reagent. Analysis was performed on a UHPLC system (Agilent 1290 series, Waldbronn, Germany) coupled with a 6460 triple-quadrupole mass spectrometer (Agilent Inc., Singapore, Singapore), by an established method with an expanded assay ability. In brief, assay was studied on a Zorbax SB-C18 column (3.0 mm × 150 mm, 5 μm, Agilent) maintaining at 50 °C. A binary solvent system consisting of methanol (A) and water (B, formic acid and 0.2% heptafluorobutyric acid) was used for gradient elution. The following gradient program was used: 0 min, 2% elute A; 1−4 min, 15% elute A; 4−5 min, 20% elute A; 9.5 min, 80% elute A; post time, 3.5 min. Flow rate was set at 0.4 mL/min and 2 μL of samples was injected. Data acquisition was carried out in the multiple reaction monitoring (MRM) mode (SI, Figs S4 and S5). The electrospray ionization (ESI) voltage was set at 5 kV. The sheath gas flow rate was 12 L/min at 350 °C. The cone gas flow rate was 10 L/min and interface temperature was 325 °C.

### Statistical analysis

The raw metabolomics data files were conducted on Agilent Quantitative Analysis version B.06.00 analyst data processing software (Agilent Corporation, USA). Data were expressed as mean ± SD in normal distribution variables. Differential metabolites among three groups were identified by the use of ANOVA analysis with a threshold of p < 0.05. Multi-group comparisons were achieved by a strict Tukey’s post-hoc test in the SPSS 11.0 software (SPSS Inc., Chicago, IL, USA). Correlation analyses were performed using the Pearson or Spearman Rank test from the R package, illustrated by heat-map. Receiver operating characteristic (ROC) analyses were performed to obtain the area under the ROC curve (AUC). Youden index was used to identify the best biomarkers cut-off level to distinguish between NA and NB patients.

## Electronic supplementary material

Supplementary Information
