# Supplementary material for: Diagnostic value of plasma tryptophan and symmetric dimethylarginine levels for acute kidney injury among tacrolimus-treated kidney transplant patients by targeted metabolomics analysis
Source: Sci Rep. 2018 Oct 2;8:14688. doi: 10.1038/s41598-018-32958-2 (PMC6168546; doi:10.1038/s41598-018-32958-2)
Supplement: Supplementary file 1 — Supplementary Information [file 41598_2018_32958_MOESM1_ESM.doc]

**Supplementary Information**

**Diagnostic value of plasma tryptophan and symmetric dimethylarginine levels for acute kidney injury among tacrolimus-treated kidney transplant patients by targeted metabolomics analysis**

Feng Zhang^1^, Qinghua Wang^2,3^, Tianyi Xia^1^, Shangxi Fu^4^, Xia Tao^1^, Yan Wen^1^, Shen’an Chan^5^, Shouhong Gao^1^, Xiaojuan Xiong^2^*, & Wansheng Chen^1^*

^1^ *Department of Pharmacy, Changzheng Hospital, Second Military Medical University, Shanghai 200003, P. R. China*

^2^ *Key Laboratory of Jiangxi Province for Research on Active Ingredients in Natural Medicines, Bioengineering Research Institute, Yichun University, Yichun, 336000, P. R. China*

^3^ *Department of Pharmacy, Xinqiao Hospital, Third Military Medical University, Chongqing 400037, P. R. China*

^4^ *Department of Organ Transplantation, Changzheng Hospital, Second Military Medical University, Shanghai 200003, P. R. China*

^5^ *Agilent Technology, Inc. Taipei, 10492, Taiwan, P. R. China*

**This file includes:**

**Text S1. Chemicals and reagents**

**Text S2. Standards and QC samples**

**Text S3. Method validation**

**Figure S1** Ion product of the detection of 25 amino acids and 3 internal standards (A: Gly, B: Ala, C: Ser, D: Pro, E: Val, F: Thr, G: AMA, H: Oxo, I: Leu, J: Iso, K: Asp, L: Gln, M: Lys, N: Glu, O: Met, P: His, Q: Phe, R: Arg, S:Cit, T: HA, U: Tyr, V: SDMA, W: Trp, X: Kyn, Y: Cys; IS-Ala: L-Alanine-d4, IS-Met: L-Methionine-d3, IS-Phe: L-Phenylalanine-d5)

**Figure S2.**  Representative MRM chromatograms of A: Gly, B: Ala, C: Ser, D: Pro, E: Val, F: Thr, G: AMA, H: Oxo, I: Iso and Leu, J: Asp, K: Gln, L: Lys, M: Glu, N: Met, O: His, Q: Phe, R: Argnine, S: Cit, T: HA, U: Tyr, V: SDMA, W: Trp, X: Kyn and Y: Cys, (IS): L-Alanine-d4, L-Methionine-d3, L-Phenylalanine-d5 before and after spiking the mixed liquor for: (1) “Mimic blank plasma”, (2) blank human plasma samples, (3) “Mimic blank plasma” added with 25 amino acids (QC in medium level) or IS (300 ng/ml).

**Figure S3** The ROC result of SDMA+Trp

**Table S1** Peak times, calibration ranges, regression equations and regression co-efficients (R) of amino acids

**Table S2** Precision and accuracy from QC samples and LLOQ of amino acids (n = 5)

**Table S3** Matrix effect and recoveries from QC samples of amino acids and IS (n = 5)

**Table S4** Stability of amino acids (concentration, μg/mL, n = 3)

**Table S5a** The correlation analysis of clinical features and 25 amino acids

**Table S5b** The correlation analysis of clinical features and 25 amino acids

**Table S6** Diagnostic performance of biomarkers.

**Text S1. Chemicals and reagents**

All the standards had the purity > 98%. L-glycine (Gly), L-alanine (Ala), L-serine (Ser), L-proline (Pro), L-valine (Val), L-threonine (Thr), L-leucine (Leu), L-isoleucine (Ile), L-aspartic acid (Asp), L-lysine (Lys), L-glutamic acid (Glu), L-methionine (Met), L-histidine (His), L-phenylalanine (Phe), L-arginin (Arg),e, L-tyrosine (Tyr), L-tryptophan (Trp), and L-cystine (Cys) were purchased from the National Institute for the Control of Pharmaceutical and Biological Products (Beijing, China). L-hippuric acid (HA), L-symmetric dimethylarginine (SDMA), L-oxoproline (Oxo), aminomalonic acid (AMA), L-glutamine (Gln), L-kynurenine (Kyn) and L-citrulline (Cit) were got from Dalian Meilun Biotech Co., Ltd (Dalian, Liaoning, China). Internal standards, including L-alanine-d4 (L-Ala-d4), L-methionine-d3 (L-Met-d3) and L-phenylalanine-d5 (L-Phe-d5) were purchased from Toronto Research Chemicals Inc (North York, Canada). Bovine serum albumin (BSA) and Phosphate Buffered Saline (PBS) were got from Shanghai Bio-Light Technology Co., Ltd. (Shanghai, China). Heptafluorobutyric acid (HFBA) was supplied by Adamas Reagent Co., Ltd. (Basel, Switzerland). Deionized water was prepared using a Milli-Q Reagent Water System (Millipore, MA, USA). All other reagents were of analytical grade or HPLC grade.

**Text S2. Standards and QC samples**

Stock solutions were made at 1 mg/mL concentrations as follows: Kyn, Asp, Arg, AMA, His, Met, Ser, Cit, Thr, Gln, Leu, Iso, Phe, Lys, Val, Gly, Oxo, Ala and Pro were prepared in 5% methanol aqueous solution; Cys, SDMA and Tyr were prepared in 4% HCl aqueous solution; Glu and Trp were prepared in 0.2% formic acid aqueous solution; and HA was prepared in methanol solution. All ISs, including L-Ala-d4, L-Met-d3 and L-Phe-d5, were prepared in 5% methanol aqueous solution. Stock solutions were then prepared by spiking 4% BSA, yielding seven concentration levels of calibration standards: 0.002, 0.005, 0.05, 0.5, 1, 2 and 3 μg/mL for Cys and HA, 0.02, 0.05, 0.1, 0.2, 0.5, 1 and 2 μg/mL for Kyn, Asp, SDMA and Arg, 0.05, 0.1, 0.25, 0.5, 1, 2 and 3μg/mL for AMA, His, Met and Ser, 0.2, 0.5, 1, 2, 4, 8 and 10 μg/mL for Glu, Thr, Cit and Gln, 0.5, 1, 2, 4, 5, 8, and 12 μg/mL for Iso, Leu, Tyr, Phe, Trp, Lys and Val, 0.5, 1, 2, 5, 10, 12 and 15 μg/mL for Gly and Oxo, and 1, 2, 5, 10, 20, 25 and 30 μg/mL for Ala and Pro. Quality control (QC) samples were prepared in the similar way: 0.005, 0.5 and 2 μg/mL for Cys and HA, 0.05, 0.25 and 1 μg/mL for Kyn, Asp, SDMA and Arg, 0.1, 0.5 and 2 μg/mL for AMA, His, Met and Ser, 0.5, 2 and 8 μg/mL for Glu, Thr, Cit and Gln, 1, 4 and 8 μg/mL for Iso, Leu, Tyr, Phe, Trp, Lys and Val, 1, 5 and 12 μg/mL for Gly and Oxo, 2, 10 and 25 μg/mL for Ala and Pro. All solutions were kept at −20 °C when not used.

**Text S3. Method validation**

The developed UHPLC-MS/MS method was validated for linearity, precision, accuracy, matrix effect, recovery, incurred sample reanalysis (ISR) and stability, according to recommendations published by FDA (US Food and Drug Administration, 2013) [R1]. The FDA guidelines required the same biological matrix as the sample matrix for method validation. However, it was hard and often unavailable to purchase amino acids-free plasma, as amino acids were endogenous compounds and varied among people. Fortunately, 4% BSA was verified as “mimic blank plasma” in the amino acids assay as an alternative strategy. Therefore, the UHPLC-MS/MS method was developed according to the currently used method [R2].

Specificity of the method was assessed by comparing the peaks of analytes in “mimic blank plasma”, blank human plasma samples, and QC in medium level. Chromatograms of these typical samples showed no other endogenous interference at the retention times of all analytes and ISs, in addition to endogenous amino acids (SI, figure S1). Linearity regression curves were obtained by plotting from the peak area ratio of each analyte to corresponding IS vs plasma concentrations using a 1/x2 weighted linear least-squares regression model. Concentrations of all analytes were calculated after correcting for endogenous ones by using SPSS 11.0 Linear Regression Model [R2]. The calibration curves were linear over the investigated ranges, with acceptable linear correlation coefficients (r ≥ 0.99) The LLOQ and LLOD were defined as signal to-noise ratio (S/N) ≥ 3:1 and 10:1, respectively, variation coefficient (CV%) of which did not exceed 20% (SI, table S1).

The inter- and intraday precisions were determined at three different levels of QC and LLOQ, with CV% ranged from 3.0% to 4.1%, and from 1.7% to 2.8%, respectively. Accuracy for the above QC samples were expressed as relative error (RE%), which values were in the range of -14.52% to 14.92%. Detailed data were shown in SI, table S2.

Matrix effect was calculated by comparing the mean area response of corrected spiked QC sample before extraction to standard solution at the three different levels. Recovery was estimated by comparing the mean area response of corrected spiked samples of pre-extraction to post-extraction. The mean matrix effect after correcting for endogenous amino acids was 89.96% (range 80.01−107.19%, except Oxo) and showed a CV of 2.98% (range 0.45−8.99%), while mean recovery was 94.02% (range 81.01−110.38%) and showed a CV of 2.95% (range 0.53−8.48%). Data were presented in SI, table S3.

An incurred sample reanalysis (ISR) was also conducted by computerized random selection of 2/3 samples, which were repeatedly measured. The obtained results were compared with the data obtained earlier, and the percent change in the value (RE) was calculated within 20% [R3].

Stability was tested by analyzing three different levels of QC by three replicate at four storage conditions: QC samples kept at room temperature for 6 h for short-term stability, extracted QC samples kept in the auto-sampler at 4 ºC for post-preparation stability, QC samples treated in three freeze-thaw cycles (from −20 ºC to 25 ºC) for freeze and thaw stability, and QC samples stored at −80 ºC for 30 days for long-term stability. The obtained results were compared with the nominal data, and the percent change in the value (RE) was calculated within 20% (SI, table S4).

**References**

1. U.S. Department of Health and Human Services Food and Drug Administration, Center for Drug Evaluation and Research: Rockville, MD. Guidance for Industry: Bioanalytical Method Validation. 2013.
2. Shu C, Zeng TM, Gao SH, Xia TY, Huang LF, Zhang F, Chen WS. LC-MS/MS method for simultaneous determination of thalidomide, lenalidomide, cyclophosphamide, bortezomib, dexamethasone and adriamycin in serum of multiple myeloma patients. J Chromatogr B Analyt Technol Biomed Life Sci. 2016, 1028, 111-119.
3. Wang QH, Wen Y, Xia TY, Xiong XJ, Gao SH, You CH, Tao X, Zhang F, Chen WS. Quantification of 18 amino acids in human plasma: application in renal transplant patient plasma by targeted UHPLC-MS/MS. Bioanalysis. 2016, 8 (13) 1337-1351.


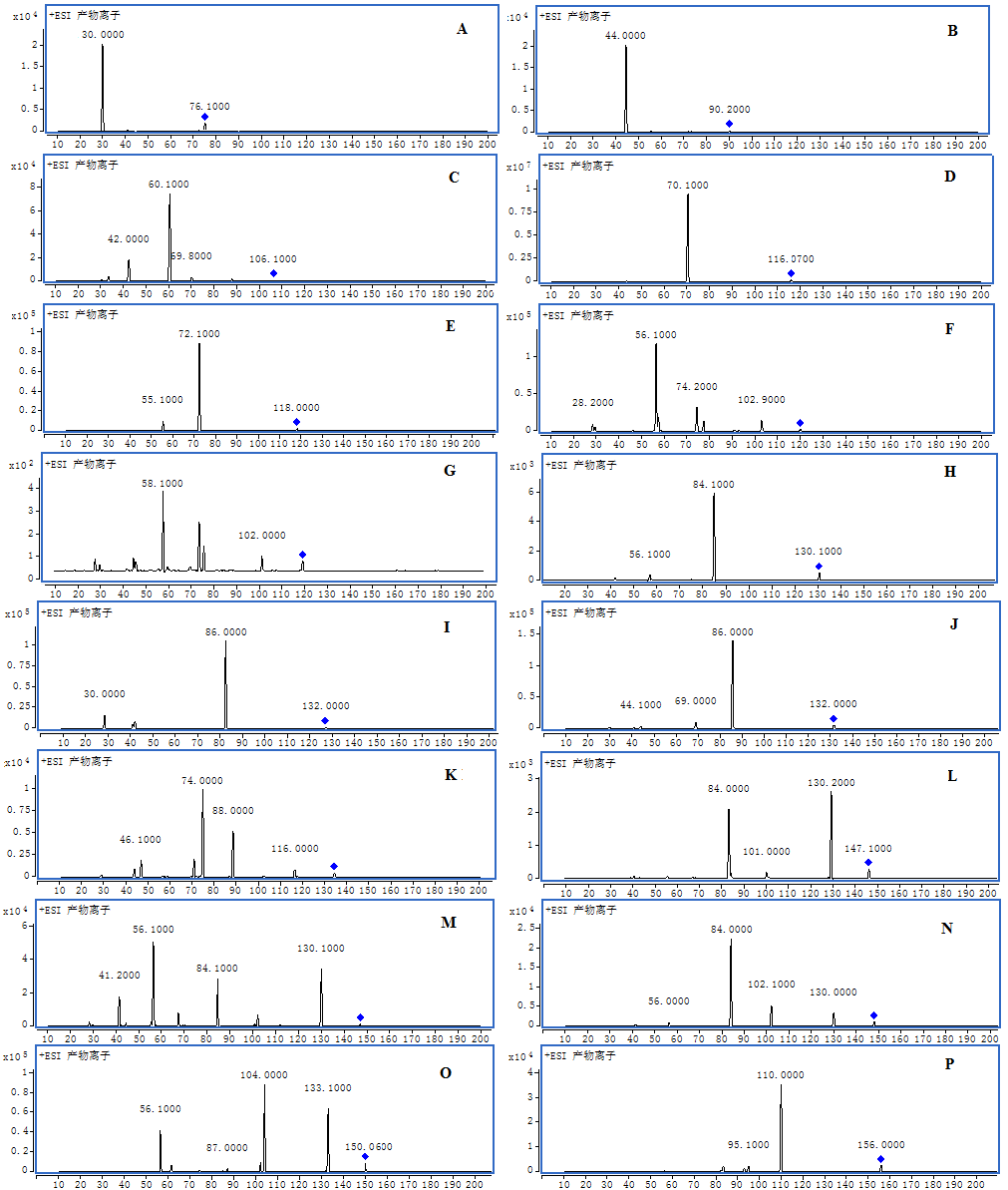


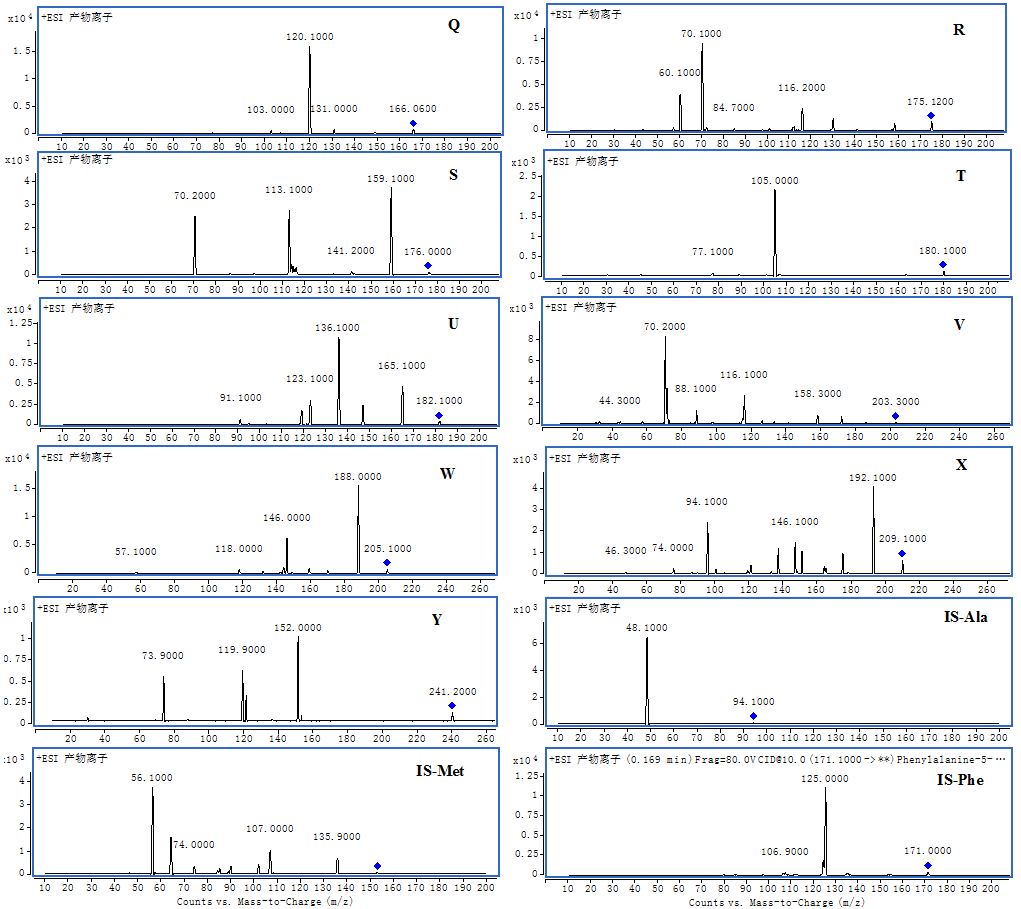


Figure S1 Ion product of the detection of 25 amino acids and 3 internal standards (A: Gly, B: Ala, C: Ser, D: Pro, E: Val, F: Thr, G: AMA, H: Oxo, I: Leu, J: Iso, K: Asp, L: Gln, M: Lys, N: Glu, O: Met, P: His, Q: Phe, R: Arg, S:Cit, T: HA, U: Tyr, V: SDMA, W: Trp, X: Kyn, Y: Cys; IS-Ala: L-Alanine-d4, IS-Met: L-Methionine-d3, IS-Phe: L-Phenylalanine-d5)


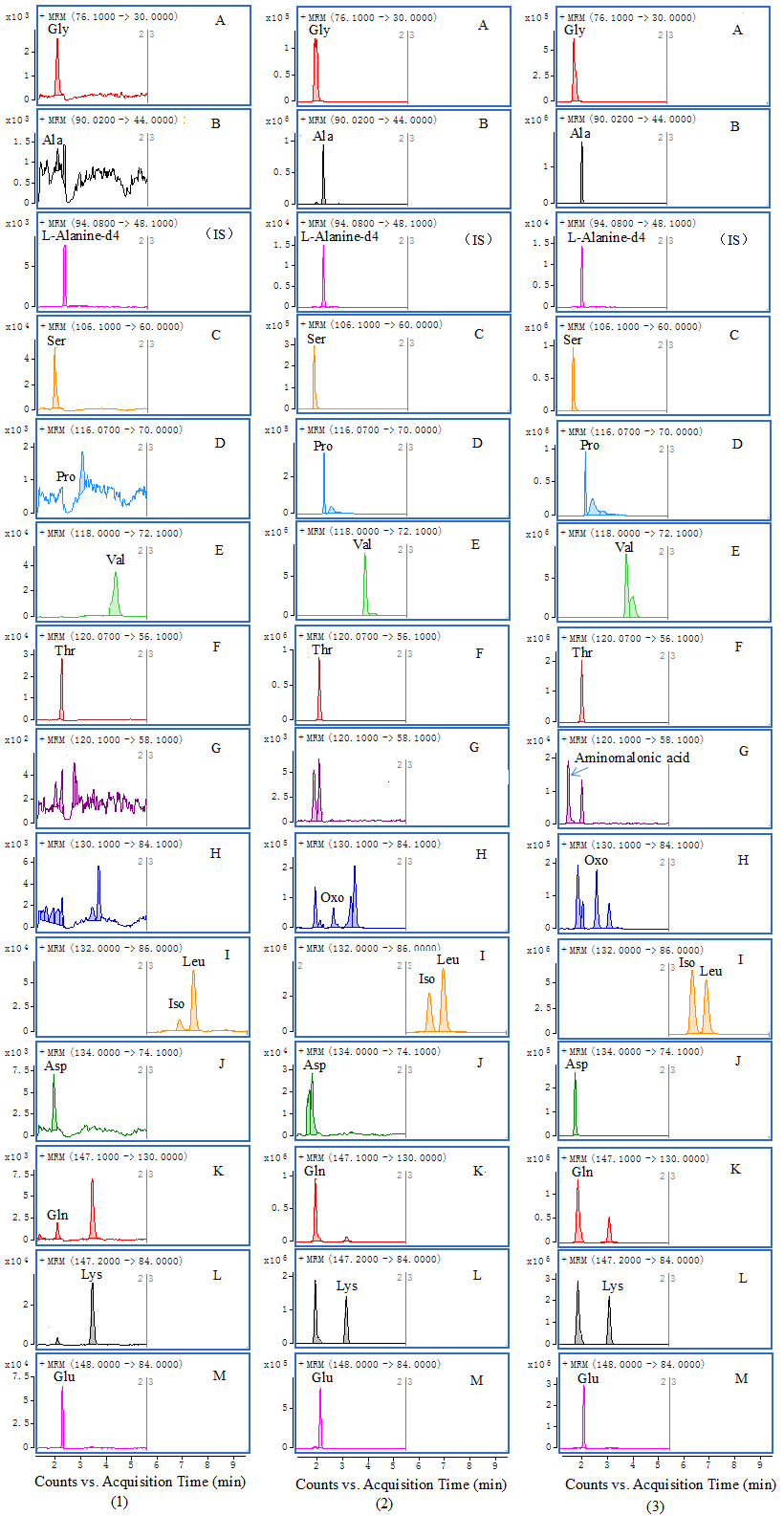


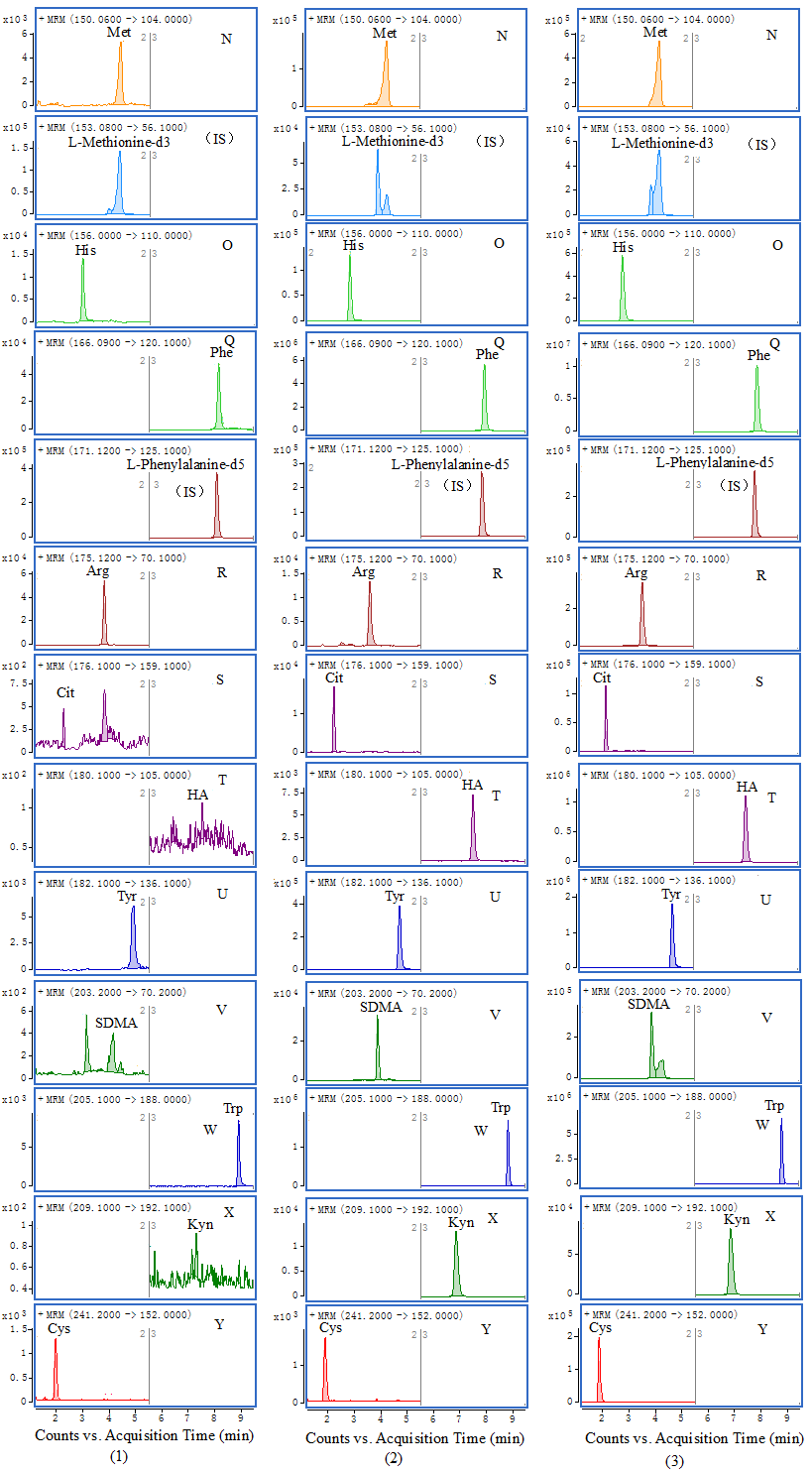


Figure S2. Representative MRM chromatograms of A: Gly, B: Ala, C: Ser, D: Pro, E: Val, F: Thr, G: AMA, H: Oxo, I: Iso and Leu, J: Asp, K: Gln, L: Lys, M: Glu, N: Met, O: His, Q: Phe, R: Argnine, S: Cit, T: HA, U: Tyr, V: SDMA, W: Trp, X: Kyn and Y: Cys, (IS): L-Alanine-d4, L-Methionine-d3, L-Phenylalanine-d5 before and after spiking the mixed liquor for: (1) “Mimic blank plasma”, (2) blank human plasma samples, (3) “Mimic blank plasma” added with 25 amino acids (QC in medium level) or IS (300 ng/ml).


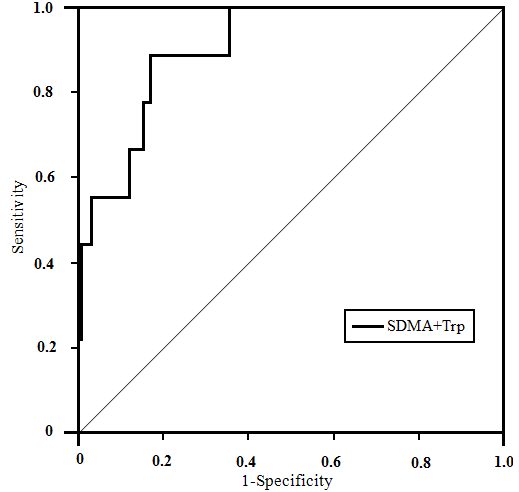


Figure S3 The ROC result of SDMA and Trp combination

Table S1 Peak times, calibration ranges, regression equations and regression co-efficients (R) of amino acids

| Analyte | Peak time  (min) | Calibration range (μg/mL) | Regression equation | *R* | LLOQ (μg/mL) | LOD  (μg/mL) |
| --- | --- | --- | --- | --- | --- | --- |
| Gly | 1.83 | 0.500 ~ 15.000 | Y=0.1897X-0.0159 | 0.9911 | 0.500 | 0.020 |
| Ala | 2.18 | 1.000 ~ 30.000 | Y=8.7414X-1.0436 | 0.9975 | 1.000 | 0.020 |
| Ser | 1.81 | 0.050 ~ 3.000 | Y=1.0398X+0.0556 | 0.9950 | 0.050 | 0.010 |
| Pro | 2.25 | 1.000 ~ 30.000 | Y=0.1686X-0.0471 | 0.9946 | 1.000 | 0.050 |
| Val | 3.85 | 0.500 ~ 12.000 | Y=7.2404X-0.2537 | 0.9989 | 0.500 | 0.010 |
| Thr | 2.09 | 0.200 ~ 10.000 | Y=0.7541X+0.1648 | 0.9958 | 0.200 | 0.020 |
| AMA | 1.54 | 0.050 ~ 3.000 | Y=0.0374X+0.0005 | 0.9954 | 0.050 | 0.020 |
| Oxo | 2.65 | 0.500 ~ 15.000 | Y=0.0277X-0.0058 | 0.9936 | 0.500 | 0.100 |
| Iso | 6.56 | 0.500 ~ 12.000 | Y=8.1045X-0.2024 | 0.9997 | 0.500 | 0.005 |
| Leu | 7.11 | 0.500 ~ 12.000 | Y=7.9342X-0.0248 | 0.9998 | 0.500 | 0.005 |
| Asp | 1.82 | 0.020 ~ 2.500 | Y=0.6094X-0.0022 | 0.9938 | 0.020 | 0.005 |
| Gln | 1.94 | 0.020 ~ 10.000 | Y=1.0903X+0.6574 | 0.9906 | 0.020 | 0.005 |
| Lys | 3.16 | 0.500 ~ 12.000 | Y=0.8972X-0.2874 | 0.9900 | 0.500 | 0.010 |
| Glu | 2.14 | 0.020 ~ 10.000 | Y=0.9084X+0.2936 | 0.9926 | 0.020 | 0.005 |
| Met | 4.29 | 0.050 ~ 3.000 | Y=4.6112X-0.0432 | 0.9973 | 0.050 | 0.002 |
| His | 2.84 | 0.050 ~ 3.000 | Y=1.0492X-0.0247 | 0.9925 | 0.050 | 0.010 |
| Phe | 7.92 | 0.500 ~ 12.000 | Y=7.9465X-0.0094 | 0.9993 | 0.500 | 0.005 |
| Arg | 3.56 | 0.020 ~ 2.500 | Y=1.1716X-0.0096 | 0.9929 | 0.020 | 0.004 |
| Cit | 2.21 | 0.020 ~ 10.000 | Y=0.0297X-0.0020 | 0.9912 | 0.020 | 0.005 |
| HA | 7.49 | 0.002 ~ 3.000 | Y=1.4556X-0.0008 | 0.9932 | 0.002 | 0.001 |
| Tyr | 4.71 | 0.500 ~ 12.000 | Y=0.6339X-0.0821 | 0.9968 | 0.500 | 0.004 |
| SDMA | 3.85 | 0.020 ~ 2.500 | Y=1.2771X-0.0118 | 0.9917 | 0.020 | 0.002 |
| Trp | 8.82 | 0.500 ~ 12.000 | Y=2.5282X-0.1412 | 0.9990 | 0.500 | 0.004 |
| Kyn | 6.96 | 0.020 ~ 2.500 | Y=0.3784X-0.0022 | 0.9918 | 0.020 | 0.002 |
| Cys | 1.85 | 0.002 ~ 3.000 | Y=0.2262X-0.0003 | 0.9933 | 0.002 | 0.001 |

Table S2 Precision and accuracy from QC samples and LLOQ of amino acids (n = 5)

| Analyte | Concentration  (μg/mL) | Intra-day | |  | Inter-day | | Analyte | Concentration  (μg/mL) | Intra-day | |  | Inter-day | |
| --- | --- | --- | --- | --- | --- | --- | --- | --- | --- | --- | --- | --- | --- |
|  |  | RSD (%) | ER (%) |  | RSD (%) | ER (%) |  |  | RSD (%) | ER (%) |  | RSD (%) | ER (%) |
| Gly | 0.500 | 1.95 | 10.20 |  | 4.75 | 7.63 | Glu | 0.200 | 5.22 | -13.70 |  | 14.13 | -0.28 |
|  | 1.000 | 7.11 | 1.18 |  | 6.93 | -0.33 |  | 0.500 | 4.22 | 8.05 |  | 6.80 | 3.87 |
|  | 5.000 | 4.10 | -6.34 |  | 3.77 | -4.28 |  | 2.000 | 4.49 | -7.63 |  | 7.67 | -9.70 |
|  | 12.000 | 4.74 | 3.76 |  | 6.80 | 3.69 |  | 8.000 | 4.96 | -10.94 |  | 6.02 | -9.72 |
| Ala | 1.000 | 2.56 | 7.02 |  | 6.60 | -0.60 | Met | 0.050 | 1.86 | 4.36 |  | 3.66 | 9.29 |
|  | 2.000 | 4.36 | -4.57 |  | 5.48 | -9.74 |  | 0.100 | 2.49 | -6.67 |  | 2.72 | -4.46 |
|  | 10.000 | 4.79 | -7.60 |  | 4.81 | -11.37 |  | 0.500 | 1.83 | -0.85 |  | 1.41 | -1.65 |
|  | 25.000 | 4.19 | -3.51 |  | 5.97 | -9.10 |  | 2.000 | 0.96 | -0.22 |  | 2.63 | 2.94 |
| Ser | 0.050 | 3.02 | 11.92 |  | 7.18 | 14.08 | His | 0.050 | 1.36 | 11.26 |  | 5.73 | 13.26 |
|  | 0.100 | 4.29 | -4.43 |  | 12.33 | 7.28 |  | 0.100 | 9.28 | 7.03 |  | 11.23 | 0.23 |
|  | 0.500 | 5.20 | 1.63 |  | 4.94 | 2.71 |  | 0.500 | 4.77 | -9.67 |  | 6.34 | -7.59 |
|  | 2.000 | 2.19 | -9.10 |  | 2.15 | -9.82 |  | 2.000 | 3.33 | 10.89 |  | 6.59 | 4.56 |
| Pro | 1.000 | 1.75 | -2.94 |  | 3.82 | -1.10 | Phe | 0.500 | 0.50 | -0.21 |  | 6.14 | 5.11 |
|  | 2.000 | 1.72 | -6.99 |  | 2.67 | -6.14 |  | 1.000 | 0.82 | -2.06 |  | 8.03 | 4.34 |
|  | 10.000 | 1.80 | -9.64 |  | 2.50 | -10.61 |  | 4.000 | 0.75 | 3.23 |  | 4.27 | 6.57 |
|  | 25.000 | 2.47 | -6.06 |  | 4.99 | -11.34 |  | 8.000 | 1.07 | -2.03 |  | 3.39 | -4.79 |
| Val | 0.500 | 1.21 | -2.60 |  | 5.99 | 5.17 | Arg | 0.020 | 4.14 | -14.30 |  | 14.94 | 3.47 |
|  | 1.000 | 1.00 | -2.81 |  | 6.49 | -0.09 |  | 0.050 | 4.52 | -14.24 |  | 10.05 | -2.51 |
|  | 4.000 | 3.71 | -3.69 |  | 3.66 | -2.20 |  | 0.500 | 2.10 | -9.93 |  | 4.75 | -4.23 |
|  | 8.000 | 1.18 | -7.64 |  | 2.37 | -5.39 |  | 2.000 | 1.07 | 4.46 |  | 4.15 | 8.36 |
| Thr | 0.200 | 8.97 | -2.83 |  | 11.37 | 7.36 | Cit | 0.200 | 10.87 | -6.51 |  | 7.75 | -4.76 |
|  | 0.500 | 4.59 | 7.91 |  | 9.54 | 14.12 |  | 0.500 | 8.24 | -12.33 |  | 7.27 | -13.42 |
|  | 2.000 | 8.94 | -4.30 |  | 9.21 | -10.58 |  | 2.000 | 0.88 | -13.92 |  | 5.34 | -14.12 |
|  | 8.000 | 2.35 | -6.38 |  | 4.98 | -0.28 |  | 8.000 | 2.59 | 5.69 |  | 10.79 | -6.72 |
| AMA | 0.050 | 12.43 | -2.75 |  | 10.37 | -2.72 | HA | 0.002 | 2.69 | 9.24 |  | 4.17 | 14.41 |
|  | 0.100 | 3.52 | -13.29 |  | 11.31 | -5.23 |  | 0.010 | 4.70 | -10.62 |  | 10.24 | -7.70 |
|  | 0.500 | 4.78 | -8.11 |  | 5.40 | -6.53 |  | 0.500 | 3.66 | 2.79 |  | 5.82 | 6.98 |
|  | 2.000 | 2.34 | -13.23 |  | 7.48 | -8.83 |  | 2.000 | 0.76 | 13.93 |  | 2.19 | 13.32 |
| Oxo | 0.500 | 5.36 | 9.16 |  | 6.97 | 7.48 | Tyr | 0.500 | 1.58 | 7.50 |  | 5.73 | 10.09 |
|  | 1.000 | 11.80 | 2.53 |  | 11.74 | 1.16 |  | 1.000 | 1.40 | -5.18 |  | 9.77 | 1.21 |
|  | 5.000 | 9.68 | -7.77 |  | 12.54 | -1.87 |  | 4.000 | 1.77 | -5.20 |  | 7.09 | -0.80 |
|  | 12.000 | 6.63 | -3.40 |  | 9.08 | 3.69 |  | 8.000 | 0.83 | 7.96 |  | 3.02 | 4.50 |
| Iso | 0.500 | 0.64 | 3.77 |  | 6.37 | 5.46 | SDMA | 0.020 | 1.16 | 8.39 |  | 3.33 | 6.47 |
|  | 1.000 | 1.12 | -2.25 |  | 8.16 | -2.29 |  | 0.050 | 3.83 | -13.94 |  | 3.59 | -13.82 |
|  | 4.000 | 2.20 | -2.25 |  | 4.80 | -0.14 |  | 0.500 | 3.93 | -10.36 |  | 6.96 | -4.81 |
|  | 8.000 | 1.69 | 5.53 |  | 6.38 | -2.79 |  | 2.000 | 1.59 | 9.53 |  | 3.68 | 10.19 |
| Leu | 0.500 | 1.05 | 1.86 |  | 6.65 | 4.43 | Trp | 0.500 | 0.71 | 4.73 |  | 4.53 | 6.92 |
|  | 1.000 | 0.96 | -3.61 |  | 7.93 | 0.90 |  | 1.000 | 0.66 | -2.14 |  | 5.44 | -0.58 |
|  | 4.000 | 1.19 | -3.58 |  | 4.34 | -1.68 |  | 4.000 | 1.23 | -1.70 |  | 2.07 | -1.74 |
|  | 8.000 | 1.22 | -3.60 |  | 2.08 | -5.99 |  | 8.000 | 0.69 | 0.55 |  | 3.09 | -1.98 |
| Asp | 0.020 | 10.04 | 2.16 |  | 13.15 | 14.96 | Kyn | 0.020 | 4.14 | 7.84 |  | 4.11 | 11.51 |
|  | 0.050 | 3.65 | -6.01 |  | 8.84 | 4.89 |  | 0.050 | 3.32 | -11.30 |  | 8.55 | -6.63 |
|  | 0.500 | 4.13 | 8.32 |  | 4.15 | 12.05 |  | 0.500 | 3.53 | -4.34 |  | 3.21 | -4.37 |
|  | 2.000 | 3.96 | -7.83 |  | 5.92 | -5.72 |  | 2.000 | 1.43 | 2.51 |  | 3.43 | 3.74 |
| Gln | 0.200 | 2.54 | 2.95 |  | 5.41 | 10.02 | Cys | 0.002 | 2.99 | 8.74 |  | 13.82 | 12.12 |
|  | 0.500 | 2.64 | 14.00 |  | 2.51 | 14.07 |  | 0.010 | 2.73 | -14.27 |  | 12.54 | -2.41 |
|  | 2.000 | 1.82 | 8.09 |  | 8.60 | 5.04 |  | 0.500 | 2.55 | 1.86 |  | 4.76 | 6.96 |
|  | 8.000 | 1.38 | -14.52 |  | 2.68 | -13.61 |  | 2.000 | 0.86 | 14.87 |  | 3.59 | 13.28 |
| Lys | 0.500 | 1.02 | 14.87 |  | 3.77 | 14.16 | — | — | — | — |  | — | — |
|  | 1.000 | 2.16 | -13.57 |  | 2.14 | -13.43 |  | — | — | — |  | — | — |
|  | 4.000 | 1.04 | -12.72 |  | 4.17 | -14.28 |  | — | — | — |  | — | — |
|  | 8.000 | 1.39 | 0.29 |  | 5.79 | 8.43 |  | — | — | — |  | — | — |

Table S3 Matrix effect and recoveries from QC samples of amino acids and IS (n = 5)

| Analyte | Concentration | Matrix effect | |  | Recovery | | Analyte | Concentration | Matrix effect | |  | Recovery | |
| --- | --- | --- | --- | --- | --- | --- | --- | --- | --- | --- | --- | --- | --- |
|  | (μg/mL) | Mean (%) | CV (%) |  | Mean (%) | CV (%) |  | (μg/mL) | Mean (%) | CV (%) |  | Mean (%) | CV (%) |
| Gly | 1.000 | 82.36 | 5.07 |  | 88.55 | 3.64 | Glu | 0.500 | 87.17 | 2.13 |  | 86.87 | 2.06 |
|  | 5.000 | 80.54 | 4.19 |  | 83.24 | 5.23 |  | 2.000 | 89.09 | 5.83 |  | 81.13 | 3.20 |
|  | 12.000 | 80.22 | 6.84 |  | 101.88 | 3.99 |  | 8.000 | 88.36 | 2.63 |  | 98.63 | 4.69 |
| Ala | 2.000 | 80.01 | 4.39 |  | 108.03 | 2.49 | Met | 0.100 | 99.36 | 1.14 |  | 88.61 | 2.20 |
|  | 10.000 | 81.04 | 4.82 |  | 96.34 | 3.84 |  | 0.500 | 95.84 | 2.43 |  | 92.48 | 2.53 |
|  | 25.000 | 88.29 | 3.61 |  | 109.96 | 3.64 |  | 2.000 | 103.55 | 1.75 |  | 104.75 | 1.36 |
| Ser | 0.100 | 86.68 | 2.31 |  | 81.87 | 5.85 | His | 0.100 | 92.96 | 8.99 |  | 87.42 | 4.58 |
|  | 0.500 | 87.40 | 2.80 |  | 89.18 | 1.39 |  | 0.500 | 89.01 | 8.67 |  | 102.30 | 8.44 |
|  | 2.000 | 82.85 | 5.59 |  | 106.86 | 2.64 |  | 2.000 | 98.69 | 6.63 |  | 97.67 | 6.91 |
| Pro | 2.000 | 81.17 | 0.57 |  | 106.66 | 3.81 | Phe | 1.000 | 85.47 | 1.29 |  | 92.73 | 0.79 |
|  | 10.000 | 82.97 | 2.98 |  | 92.47 | 3.82 |  | 4.000 | 84.14 | 1.95 |  | 96.79 | 1.19 |
|  | 25.000 | 86.18 | 3.10 |  | 110.24 | 4.91 |  | 8.000 | 86.21 | 0.86 |  | 100.37 | 0.81 |
| Val | 1.000 | 97.34 | 0.97 |  | 86.70 | 0.69 | Arg | 0.050 | 95.93 | 1.83 |  | 84.89 | 3.98 |
|  | 4.000 | 87.38 | 3.36 |  | 90.54 | 1.94 |  | 0.500 | 92.44 | 1.84 |  | 85.73 | 2.38 |
|  | 8.000 | 104.64 | 1.46 |  | 103.90 | 2.16 |  | 2.000 | 97.40 | 0.71 |  | 94.06 | 1.17 |
| Thr | 0.500 | 86.70 | 1.23 |  | 91.52 | 2.44 | Cit | 0.500 | 93.42 | 8.01 |  | 105.02 | 7.90 |
|  | 2.000 | 81.05 | 3.50 |  | 83.87 | 2.85 |  | 2.000 | 87.96 | 3.14 |  | 86.27 | 5.16 |
|  | 8.000 | 82.28 | 3.16 |  | 100.45 | 2.54 |  | 8.000 | 93.85 | 6.91 |  | 110.38 | 3.95 |
| AMA | 0.100 | 87.00 | 4.67 |  | 82.26 | 4.86 | HA | 0.010 | 98.21 | 3.20 |  | 95.93 | 3.80 |
|  | 0.500 | 87.41 | 2.67 |  | 87.02 | 1.81 |  | 0.500 | 96.56 | 1.90 |  | 105.05 | 1.74 |
|  | 2.000 | 89.18 | 1.53 |  | 87.27 | 2.95 |  | 2.000 | 96.43 | 1.15 |  | 102.87 | 1.48 |
| Oxo | 1.000 | 4.77 | 4.67 |  | 106.77 | 4.60 | Tyr | 1.000 | 88.14 | 1.61 |  | 89.52 | 1.35 |
|  | 5.000 | 8.17 | 6.83 |  | 87.95 | 4.96 |  | 4.000 | 83.56 | 2.78 |  | 92.94 | 1.91 |
|  | 12.000 | 17.93 | 6.12 |  | 101.41 | 8.48 |  | 8.000 | 91.52 | 1.44 |  | 81.11 | 4.95 |
| Iso | 1.000 | 95.22 | 1.12 |  | 89.79 | 0.88 | SDMA | 0.050 | 102.89 | 1.49 |  | 84.57 | 1.35 |
|  | 4.000 | 88.89 | 3.93 |  | 96.96 | 1.43 |  | 0.500 | 92.03 | 0.98 |  | 89.29 | 2.52 |
|  | 8.000 | 96.65 | 1.10 |  | 101.53 | 1.59 |  | 2.000 | 106.01 | 1.30 |  | 102.33 | 1.03 |
| Leu | 1.000 | 94.81 | 0.89 |  | 91.33 | 0.97 | Trp | 1.000 | 93.56 | 1.29 |  | 92.53 | 1.23 |
|  | 4.000 | 88.78 | 3.70 |  | 98.10 | 1.83 |  | 4.000 | 92.60 | 2.23 |  | 98.58 | 0.97 |
|  | 8.000 | 96.97 | 1.60 |  | 102.28 | 1.57 |  | 8.000 | 97.00 | 0.91 |  | 99.80 | 0.90 |
| Asp | 0.050 | 87.50 | 1.82 |  | 83.15 | 4.02 | Kyn | 0.050 | 86.80 | 1.42 |  | 96.35 | 4.00 |
|  | 0.500 | 85.48 | 4.37 |  | 86.06 | 3.71 |  | 0.500 | 87.26 | 1.69 |  | 96.33 | 2.38 |
|  | 2.000 | 83.32 | 3.09 |  | 88.96 | 2.02 |  | 2.000 | 94.82 | 1.06 |  | 99.26 | 0.78 |
| Gln | 0.500 | 88.59 | 2.46 |  | 89.67 | 2.07 | Cys | 0.010 | 82.08 | 3.54 |  | 82.95 | 6.37 |
|  | 2.000 | 80.11 | 2.46 |  | 88.88 | 3.16 |  | 0.500 | 80.42 | 2.28 |  | 81.61 | 2.58 |
|  | 8.000 | 89.83 | 3.52 |  | 98.99 | 3.08 |  | 2.000 | 89.53 | 4.45 |  | 81.09 | 3.09 |
| Lys | 1.000 | 107.19 | 0.45 |  | 81.01 | 1.39 | L-Ala-d4 | 0.300 | 88.45 | 4.42 |  | 107.27 | 3.70 |
|  | 4.000 | 84.97 | 3.84 |  | 81.29 | 3.04 | L-Met-d3 | 0.300 | 89.01 | 2.25 |  | 99.54 | 0.81 |
|  | 8.000 | 84.70 | 2.64 |  | 93.92 | 3.33 | L-Phe-d5 | 0.300 | 93.58 | 1.18 |  | 101.52 | 0.53 |

Table S4 Stability of amino acids (concentration, μg/mL, n = 3)

| Analyte | Concentration | Short-term stability | |  | Long-term stability | |  | Freeze and thaw stability | |  | Post-preparative stability | |
| --- | --- | --- | --- | --- | --- | --- | --- | --- | --- | --- | --- | --- |
|  |  | Detected  (Mean ± S.D) | RE (%) |  | Detected  (Mean ± S.D.) | RE (%) |  | Detected  (Mean ± S.D.) | RE (%) |  | Detected  (Mean ± S.D.) | RE (%) |
| Gly | 1.000 | 0.921±0.04 | -7.91 |  | 1.001±0.10 | 0.11 |  | 0.897±0.03 | -10.34 |  | 0.917±0.04 | -8.34 |
|  | 5.000 | 4.296±0.14 | -14.09 |  | 4.556±0.33 | -8.88 |  | 4.407±0.11 | -11.87 |  | 4.381±0.11 | -12.37 |
|  | 12.000 | 12.136±0.30 | 1.13 |  | 12.785±0.98 | 6.54 |  | 12.026±0.56 | 0.22 |  | 12.030±0.30 | 0.25 |
| Ala | 2.000 | 1.967±0.10 | -1.67 |  | 2.147±0.12 | 7.37 |  | 1.963±0.17 | -1.86 |  | 2.001±0.09 | 0.06 |
|  | 10.000 | 9.504±0.71 | -4.96 |  | 10.639±0.87 | 6.39 |  | 9.548±0.85 | -4.52 |  | 9.634±0.58 | -3.66 |
|  | 25.000 | 24.931±1.10 | -0.28 |  | 25.413±1.49 | 1.65 |  | 24.973±1.76 | -0.11 |  | 24.475±1.50 | -2.10 |
| Ser | 0.100 | 0.087±0.01 | -13.15 |  | 0.101±0.01 | 1.24 |  | 0.097±0.01 | -2.79 |  | 0.086±0.01 | -13.80 |
|  | 0.500 | 0.434±0.04 | -13.13 |  | 0.497±0.09 | -0.65 |  | 0.454±0.05 | -9.20 |  | 0.441±0.04 | -11.82 |
|  | 2.000 | 1.777±0.09 | -11.17 |  | 2.025±0.21 | 1.27 |  | 1.823±0.08 | -8.86 |  | 1.783±0.10 | -10.86 |
| Pro | 2.000 | 1.892±0.03 | -5.39 |  | 2.082±0.22 | 4.08 |  | 1.907±0.11 | -4.63 |  | 1.903±0.08 | -4.83 |
|  | 10.000 | 9.357±0.46 | -6.43 |  | 10.470±0.85 | 4.70 |  | 9.655±0.65 | -3.45 |  | 10.072±0.44 | 0.72 |
|  | 25.000 | 26.265±0.30 | 5.06 |  | 27.333±1.35 | 9.41 |  | 26.307±1.27 | 5.23 |  | 26.551±0.59 | 6.21 |
| Val | 1.000 | 0.922±0.02 | -7.85 |  | 1.030±0.10 | 2.99 |  | 0.901±0.02 | -9.88 |  | 0.897±0.03 | -10.27 |
|  | 4.000 | 3.554±0.14 | -11.16 |  | 3.958±0.57 | -1.04 |  | 3.464±0.10 | -13.40 |  | 3.439±0.06 | -14.02 |
|  | 8.000 | 7.739±0.21 | -3.27 |  | 8.500±0.72 | 6.25 |  | 7.638±0.18 | -4.52 |  | 7.510±0.35 | -6.12 |
| Thr | 0.500 | 0.478±0.05 | -4.44 |  | 0.539±0.03 | 7.88 |  | 0.467±0.06 | -6.65 |  | 0.464±0.04 | -7.26 |
|  | 2.000 | 1.713±0.09 | -14.35 |  | 2.014±0.25 | 0.68 |  | 2.148±0.77 | 7.41 |  | 1.740±0.07 | -12.99 |
|  | 8.000 | 7.919±0.10 | -1.01 |  | 8.544±0.63 | 6.81 |  | 7.668±0.39 | -4.15 |  | 7.421±0.45 | -7.23 |
| AMA | 0.100 | 0.091±0.01 | -8.75 |  | 0.098±0.01 | -2.06 |  | 0.087±0.00 | -12.56 |  | 0.092±0.01 | -8.46 |
|  | 0.500 | 0.441±0.02 | -11.72 |  | 0.472±0.03 | -5.61 |  | 0.439±0.02 | -12.22 |  | 0.452±0.02 | -9.63 |
|  | 2.000 | 1.742±0.07 | -12.89 |  | 2.033±0.33 | 1.64 |  | 1.754±0.07 | -12.30 |  | 1.742±0.08 | -12.88 |
| Oxo | 1.000 | 0.886±0.07 | -11.42 |  | 0.976±0.09 | -2.43 |  | 0.955±0.05 | -4.53 |  | 0.921±0.07 | -7.85 |
|  | 5.000 | 4.619±0.24 | -7.61 |  | 4.876±0.29 | -2.47 |  | 4.525±0.31 | -9.51 |  | 4.414±0.29 | -11.72 |
|  | 12.000 | 10.947±0.46 | -8.78 |  | 12.230±1.69 | 1.91 |  | 11.155±0.77 | -7.04 |  | 11.271±1.02 | -6.08 |
| Iso | 1.000 | 1.045±0.08 | 4.46 |  | 1.036±0.07 | 3.63 |  | 1.046±0.10 | 4.56 |  | 1.065±0.08 | 6.47 |
|  | 4.000 | 3.971±0.45 | -0.74 |  | 4.070±0.57 | 1.76 |  | 3.935±0.46 | -1.63 |  | 3.856±0.27 | -3.59 |
|  | 8.000 | 8.333±0.60 | 4.16 |  | 8.512±0.81 | 6.40 |  | 8.403±0.86 | 5.03 |  | 8.440±0.57 | 5.50 |
| Leu | 1.000 | 0.979±0.01 | -2.15 |  | 0.962±0.07 | -3.80 |  | 0.981±0.04 | -1.93 |  | 0.985±0.04 | -1.51 |
|  | 4.000 | 3.791±0.16 | -5.23 |  | 3.652±0.15 | -8.70 |  | 3.765±0.31 | -5.87 |  | 3.686±0.13 | -7.85 |
|  | 8.000 | 7.919±0.19 | -1.02 |  | 7.781±0.48 | -2.74 |  | 7.970±0.42 | -0.37 |  | 7.894±0.26 | -1.32 |
| Asp | 0.050 | 0.044±0.00 | -12.91 |  | 0.049±0.01 | -2.81 |  | 0.049±0.01 | -2.34 |  | 0.043±0.00 | -14.15 |
|  | 0.500 | 0.475±0.04 | -5.00 |  | 0.459±0.03 | -8.24 |  | 0.433±0.03 | -13.47 |  | 0.428±0.01 | -14.33 |
|  | 2.000 | 1.897±0.05 | -5.15 |  | 2.140±0.24 | 6.98 |  | 1.803±0.09 | -9.84 |  | 1.839±0.11 | -8.03 |
| Gln | 0.500 | 0.539±0.01 | 7.84 |  | 0.534±0.01 | 6.89 |  | 0.540±0.02 | 8.01 |  | 0.535±0.01 | 7.10 |
|  | 2.000 | 2.133±0.06 | 6.64 |  | 2.146±0.10 | 7.29 |  | 2.051±0.06 | 2.56 |  | 1.949±0.13 | -2.57 |
|  | 8.000 | 7.000±0.11 | -12.50 |  | 7.920±0.94 | -1.00 |  | 6.810±0.19 | -14.88 |  | 6.949±0.12 | -13.14 |
| Lys | 1.000 | 0.861±0.03 | -13.91 |  | 0.899±0.07 | -10.09 |  | 0.857±0.02 | -14.31 |  | 0.854±0.02 | -14.63 |
|  | 4.000 | 3.434±0.08 | -14.16 |  | 3.673±0.27 | -8.18 |  | 3.460±0.12 | 13.51 |  | 3.489±0.11 | -12.76 |
|  | 8.000 | 8.011±0.29 | 0.14 |  | 8.419±0.74 | 5.24 |  | 7.553±0.47 | -5.59 |  | 7.097±0.51 | -11.29 |
| Glu | 0.500 | 0.438±0.03 | -12.34 |  | 0.506±0.06 | 1.10 |  | 0.451±0.03 | -9.74 |  | 0.442±0.03 | -11.69 |
|  | 2.000 | 1.779±0.07 | -11.07 |  | 1.771±0.05 | -11.45 |  | 1.726±0.05 | -13.71 |  | 1.730±0.05 | -13.51 |
|  | 8.000 | 7.099±0.34 | -11.26 |  | 8.234±0.98 | 2.92 |  | 7.078±0.43 | -11.52 |  | 7.368±0.60 | -7.90 |
| Met | 0.100 | 0.093±0.00 | -6.86 |  | 0.103±0.01 | 3.19 |  | 0.094±0.01 | -6.43 |  | 0.092±0.00 | -8.40 |
|  | 0.500 | 0.485±0.01 | -3.01 |  | 0.522±0.05 | 4.34 |  | 0.472±0.01 | -5.56 |  | 0.472±0.01 | -5.66 |
|  | 2.000 | 2.072±0.06 | 3.60 |  | 2.050±0.07 | 2.50 |  | 2.071±0.06 | 3.56 |  | 2.037±0.03 | 1.87 |
| His | 0.100 | 0.103±0.01 | 2.88 |  | 0.105±0.01 | 4.77 |  | 0.102±0.01 | 2.32 |  | 0.097±0.01 | -3.22 |
|  | 0.500 | 0.445±0.02 | -10.91 |  | 0.436±0.02 | -12.82 |  | 0.431±0.01 | -13.86 |  | 0.433±0.01 | -13.46 |
|  | 2.000 | 1.961±0.22 | -1.96 |  | 1.994±0.26 | -0.28 |  | 1.906±0.18 | -4.72 |  | 1.877±0.14 | -6.13 |
| Phe | 1.000 | 1.015±0.01 | 1.47 |  | 1.054±0.06 | 5.38 |  | 1.005±0.01 | 0.52 |  | 1.014±0.01 | 1.42 |
|  | 4.000 | 4.157±0.08 | 3.92 |  | 4.105±0.08 | 2.63 |  | 4.151±0.05 | 3.78 |  | 4.113±0.04 | 2.83 |
|  | 8.000 | 8.121±0.12 | 1.51 |  | 8.385±0.65 | 4.81 |  | 7.978±0.07 | -0.28 |  | 7.965±0.11 | -0.44 |
| Arg | 0.050 | 0.044±0.00 | -12.71 |  | 0.046±0.00 | -7.78 |  | 0.045±0.00 | -9.23 |  | 0.045±0.00 | -10.03 |
|  | 0.500 | 0.443±0.02 | -11.33 |  | 0.465±0.03 | -7.07 |  | 0.429±0.02 | -14.15 |  | 0.428±0.01 | -14.36 |
|  | 2.000 | 2.109±0.03 | 5.46 |  | 2.284±0.22 | 14.20 |  | 2.015±0.12 | 0.75 |  | 1.929±0.12 | -3.55 |
| Cit | 0.500 | 0.435±0.03 | -13.02 |  | 0.498±0.06 | -0.31 |  | 0.453±0.04 | -9.43 |  | 0.451±0.03 | -9.77 |
|  | 2.000 | 1.770±0.06 | -11.51 |  | 1.841±0.10 | -7.96 |  | 1.925±0.21 | -3.73 |  | 1.875±0.22 | -6.23 |
|  | 8.000 | 8.496±0.79 | 6.20 |  | 8.558±0.84 | 6.98 |  | 8.306±1.00 | 3.82 |  | 8.147±0.68 | 1.83 |
| HA | 0.010 | 0.009±0.00 | 13.12 |  | 0.009±0.00 | -14.67 |  | 0.009±0.00 | -12.65 |  | 0.009±0.00 | -11.31 |
|  | 0.500 | 0.537±0.03 | 7.49 |  | 0.514±0.04 | 2.77 |  | 0.540±0.03 | 7.91 |  | 0.541±0.03 | 8.24 |
|  | 2.000 | 2.230±0.09 | 11.49 |  | 2.164±0.12 | 8.22 |  | 2.234±0.06 | 11.69 |  | 2.227±0.08 | 11.35 |
| Tyr | 1.000 | 0.990±0.04 | -0.97 |  | 0.942±0.04 | -5.83 |  | 0.972±0.04 | -2.85 |  | 0.988±0.03 | -1.21 |
|  | 4.000 | 3.779±0.33 | -5.53 |  | 3.715±0.26 | -7.11 |  | 3.640±0.21 | -9.00 |  | 3.597±0.10 | -10.07 |
|  | 8.000 | 8.525±0.31 | 6.56 |  | 8.642±0.51 | 8.03 |  | 8.441±0.33 | 5.52 |  | 8.435±0.24 | 5.44 |
| SDMA | 0.050 | 0.044±0.00 | -12.26 |  | 0.048±0.00 | -4.04 |  | 0.043±0.00 | -14.33 |  | 0.044±0.00 | -12.80 |
|  | 0.500 | 0.451±0.03 | -9.79 |  | 0.455±0.03 | -8.98 |  | 0.433±0.03 | -13.30 |  | 0.436±0.03 | -12.75 |
|  | 2.000 | 2.079±0.09 | 3.95 |  | 2.052±0.07 | 2.58 |  | 1.969±0.16 | -1.53 |  | 1.903±0.10 | -4.84 |
| Trp | 1.000 | 0.989±0.04 | -1.09 |  | 0.992±0.06 | -0.76 |  | 0.976±0.03 | -2.38 |  | 0.996±0.02 | -0.39 |
|  | 4.000 | 4.049±0.15 | 1.22 |  | 4.166±0.18 | 4.32 |  | 4.061±0.09 | 1.53 |  | 4.060±0.10 | 1.50 |
|  | 8.000 | 8.253±0.09 | 3.16 |  | 8.023±0.35 | 0.29 |  | 8.215±0.12 | 2.69 |  | 8.212±0.08 | 2.65 |
| Kyn | 0.050 | 0.045±0.00 | -10.82 |  | 0.050±0.01 | 0.92 |  | 0.044±0.00 | -11.78 |  | 0.044±0.00 | -11.51 |
|  | 0.500 | 0.471±0.02 | -5.72 |  | 0.523±0.04 | 4.59 |  | 0.467±0.02 | -6.68 |  | 0.464±0.02 | -7.26 |
|  | 2.000 | 2.173±0.04 | 8.65 |  | 2.205±0.02 | 10.25 |  | 2.138±0.07 | 6.91 |  | 2.129±0.06 | 6.43 |
| Cys | 0.010 | 0.009±0.00 | -12.60 |  | ND | — |  | 0.009±0.00 | -12.23 |  | 0.009±0.00 | -13.85 |
|  | 0.500 | 0.472±0.05 | -5.60 |  | ND | — |  | 0.438±0.02 | -12.40 |  | 0.433±0.02 | -13.40 |
|  | 2.000 | 2.139±0.17 | 6.95 |  | ND | — |  | 2.037±0.12 | 1.86 |  | 1.944±0.05 | -2.78 |

“ND” means “Not Detected”.

Table S5a The correlation analysis of clinical features and 25 amino acids

| Analyte | *Scr* | *eGFR* | FK506 C_0_ | *BUN* | UA | GLU | ALB | TP | GLB |
| --- | --- | --- | --- | --- | --- | --- | --- | --- | --- |
| Gly | *0.194* | *-0.263* | -0.359* | *0.035* | 0.110 | 0.103 | -0.423** | -0.075 | 0.412* |
| Ala | *0.276* | *-0.179* | 0.050 | *-0.061* | 0.306 | 0.000 | -0.269 | -0.040 | 0.301 |
| Ser | *0.168* | *-0.272* | -0.473** | *0.138* | -0.006 | -0.251 | -0.117 | -0.191 | -0.117 |
| Pro | *0.224* | *-0.168* | -0.314* | *0.025* | 0.184 | 0.037 | -0.395* | -0.073 | 0.360* |
| Val | *-0.053* | *0.087* | 0.450** | *0.096* | -0.107 | 0.080 | 0.347* | 0.083 | -0.296 |
| Thr | *0.139* | *-0.199* | -0.555** | *-0.056* | -0.003 | -0.212 | -0.286 | -0.277 | -0.038 |
| Oxo | *0.126* | *-0.080* | 0.055 | *-0.031* | 0.027 | 0.104 | -0.335* | -0.186 | 0.162 |
| Leu | *0.063* | *-0.037* | 0.185 | *0.150* | -0.039 | 0.028 | 0.450** | 0.261 | -0.151 |
| Iso | *0.276* | *-0.244* | 0.079 | *0.299* | 0.189 | -0.095 | 0.472** | 0.287 | -0.151 |
| Asp | -0.148 | 0.219 | 0.272 | 0.077 | -0.303 | 0.190 | -0.064 | -0.018 | 0.108 |
| Gln | *0.253* | *-0.342** | -0.405** | *0.191* | 0.113 | -0.315 | 0.047 | -0.115 | -0.228 |
| Lys | *0.128* | *-0.254* | 0.008 | *0.227* | -0.094 | 0.004 | 0.094 | -0.091 | -0.215 |
| Glu | *0.172* | *-0.088* | -0.228 | *-0.162* | -0.004 | 0.007 | -0.047 | -0.126 | -0.124 |
| Met | *0.043* | *-0.031* | 0.142 | *0.085* | 0.125 | 0.195 | 0.139 | 0.132 | 0.022 |
| His | *-0.138* | *0.175* | 0.606** | *0.195* | -0.315* | 0.399** | 0.083 | 0.063 | -0.015 |
| Phe | *0.193* | *-0.168* | 0.122 | *0.085* | 0.214 | 0.444** | 0.128 | 0.353* | 0.355* |
| Arg | *0.153* | *-0.237* | 0.441** | *0.232* | 0.048 | -0.054 | 0.038 | -0.096 | -0.197 |
| Cit | *0.512*** | *-0.539*** | *-0.510*** | *0.347** | *0.408*** | 0.005 | -0.351* | -0.166 | 0.183 |
| HA | *-0.025* | *0.037* | *-0.335** | *0.100* | *-0.087* | *-0.282* | *-0.229* | *-0.165* | *0.012* |
| Tyr | *0.015* | *0.045* | 0.064 | *-0.009* | 0.143 | 0.268 | 0.046 | 0.426** | 0.538** |
| SDMA | *0.646*** | *-0.665*** | *-0.405** | *0.560*** | *0.602*** | *-0.082* | *-0.465*** | *-0.484*** | *-0.304** |
| Trp | *-0.668*** | *0.669*** | 0.325* | *-0.640** | -0.656* | -0.203 | 0.619** | 0.513** | -0.007 |
| Kyn | *0.104* | *-0.018* | 0.092 | *0.191* | 0.469** | 0.499** | -0.038 | 0.249 | 0.377* |
| Cys | *0.481*** | *-0.534*** | *-0.498*** | *0.424*** | *0.501*** | *0.285* | *-0.251* | *-0.047* | *0.161* |
| Leu/Iso | *-0.338** | *0.436*** | 0.059 | *-0.399** | -0.260 | 0.344* | -0.188 | -0.053 | 0.157 |
| Gln/Glu | *0.028* | *-0.132* | 0.083 | *0.161* | 0.208 | 0.093 | 0.169 | 0.145 | -0.007 |
| ALB/ Leu | *-0.203* | *0.158* | *-0.159* | *-0.297** | *-0.095* | *0.196* | *0.511*** | *0.506*** | *0.267** |
| ALB/ Iso | *-0.369*** | *0.329** | *-0.084* | *-0.407*** | *-0.001* | *0.243* | *0.460*** | *0.465*** | *0.228* |
| ALB /Val | *-0.070* | *0.062* | *-0.108* | *-0.180* | *0.008* | *0.066* | *0.534*** | *0.550*** | *0.349*** |
| Pro/Cit | *-0.428*** | *0.518*** | *0.380** | *-0.326** | *-0.372** | *-0.221* | *0.069* | *0.149* | *0.285* |
| Phe/Tyr | *0.201* | *-0.248* | -0.155 | *0.105* | 0.053 | 0.019 | 0.076 | -0.141 | -0.277 |
| Kyn/Trp | *0.335** | *-0.231* | -0.055 | *0.319** | 0.468** | 0.416** | -0.253 | -0.013 | 0.264 |
| Arg/Phe | *0.070* | *-0.157* | -0.168 | *0.182* | 0.012 | -0.152 | -0.020 | -0.156 | -0.214 |
| SDMA/SCr | *-0.671*** | *0.662*** | *-0.026* | *-0.539*** | *-0.452*** | *0.056* | *-0.232* | *-0.224* | *-0.108* |
| Ser/Gly | *0.084* | *-0.127* | -0.246 | *0.220* | -0.093 | -0.385* | 0.201 | -0.055 | 0.317 |
| Val/Gly | *-0.167* | *0.241* | 0.483** | *-0.023* | -0.054 | -0.307 | 0.432** | 0.171 | -0.279 |
| Arg/Cit | *-0.511*** | *0.481*** | 0.454** | *-0.023* | -0.395* | 0.222 | 0.333* | 0.419* | 0.177 |
| TAA | *0.387** | *-0.363** | -0.371** | *0.252* | 0.239 | 0.029 | -0.358* | -0.370* | 0.479** |
| TEAA | *0.041* | *-0.074* | 0.087 | *0.100* | -0.028 | 0.030 | 0.427** | 0.256 | -0.128 |
| TNEAA | *0.375** | *-0.343** | -0.405** | *0.209* | 0.252 | 0.024 | -0.394* | -0.117 | 0.317 |
| TBCAA | *0.056* | *-0.022* | 0.202 | *0.158* | -0.011 | 0.020 | 0.473** | 0.226 | -0.235 |
| TAAA | *-0.080* | *0.117* | 0.248 | *-0.118* | 0.038 | 0.256 | 0.405* | 0.642** | 0.428** |
| TGAA | *0.333** | *-0.422** | -0.433** | *0.149* | 0.246 | -0.021 | -0.342* | -0.099 | 0.280 |
| TKAA | *0.048* | *-0.078* | 0.053 | *0.033* | 0.017 | 0.044 | 0.364* | 0.343* | 0.072 |
| TGKAA | *0.021* | *-0.022* | -0.005 | *-0.056* | 0.086 | 0.053 | 0.305 | 0.446** | 0.280 |
| TBCAA/Tyr | *0.138* | *-0.170* | -0.155 | *0.157* | -0.084 | -0.253 | 0.204 | -0.240 | -0.574** |
| ALB/TBAA | *0.236* | *-0.143* | *-0.037* | *-0.125* | *0.165* | *0.112* | *-0.228* | *-0.299* | *0.173* |
| TEAA/TNEAA | *-0.354** | *0.387** | 0.492** | *-0.352** | -0.224 | 0.114 | 0.576** | 0.330* | -0.224 |

* *P*<0.05, ** *P*<0.01.

Italic numbers with underline indicate the Spearman correlation (ρ), and the normal numbers indicate the Pearson correlation (r).

Table S5b The correlation analysis of clinical features and 25 amino acids

| Analyte | A/G | WBC | HGB | RBC | LYM | PLT | LDH | *VC* | *U-pH* |
| --- | --- | --- | --- | --- | --- | --- | --- | --- | --- |
| Gly | -0.472** | -0.155 | -0.203 | -0.187 | -0.205 | 0.198 | -0.121 | *0.219* | *0.243* |
| Ala | -0.328* | -0.114 | -0.101 | -0.130 | -0.096 | 0.275 | 0.090 | *-0.011* | *0.322* |
| Ser | 0.026 | 0.056 | -0.119 | -0.140 | 0.125 | 0.122 | -0.304 | *0.137* | *0.232* |
| Pro | -0.428** | -0.332* | -0.168 | -0.154 | -0.224 | 0.204 | -0.049 | *-0.019* | *0.266* |
| Val | 0.356* | 0.133 | 0.418** | 0.411* | -0.052 | -0.306 | 0.380 | *-0.063* | *-0.184* |
| Thr | -0.117 | -0.077 | -0.060 | -0.082 | 0.071 | 0.006 | -0.229 | *-0.041* | *0.347** |
| Oxo | -0.252 | 0.011 | -0.068 | -0.047 | -0.230 | 0.017 | -0.073 | *0.382** | *0.315* |
| Leu | 0.275 | 0.487** | 0.531** | 0.476** | 0.155 | 0.089 | 0.173 | *-0.152* | *0.028* |
| Iso | 0.282 | 0.399* | 0.400* | 0.341* | 0.086 | 0.158 | 0.178 | *-0.085* | *-0.081* |
| Asp | *-0.138* | *0.246* | *0.148* | *0.108* | *-0.058* | *-0.187* | *0.261* | *-0.145* | *-0.167* |
| Gln | 0.187 | 0.089 | 0.041 | 0.019 | 0.059 | -0.070 | -0.279 | *-0.019* | *-0.018* |
| Lys | 0.174 | 0.391* | -0.070 | -0.078 | 0.178 | 0.191 | 0.011 | *0.078* | *-0.017* |
| Glu | 0.122 | -0.225 | -0.036 | -0.034 | -0.108 | -0.321 | -0.058 | *0.167* | *0.459*** |
| Met | 0.000 | 0.352* | 0.035 | 0.011 | -0.036 | 0.354* | 0.664** | *0.411** | *-0.120* |
| His | -0.019 | 0.351* | -0.084 | -0.101 | -0.087 | -0.002 | 0.482* | *-0.004* | *-0.398** |
| Phe | -0.160 | -0.011 | -0.095 | -0.052 | -0.243 | 0.226 | 0.476* | *0.137* | *0.098* |
| Arg | 0.133 | 0.016 | -0.216 | -0.210 | 0.030 | 0.119 | 0.241 | *-0.204* | *-0.207* |
| Cit | -0.258 | -0.430** | -0.347* | -0.322 | 0.293 | 0.114 | -0.030 | *0.123* | *0.076* |
| HA | *-0.156* | *-0.024* | *0.050* | *0.009* | *0.165* | *-0.031* | *-0.275* | *-0.204* | *-0.145* |
| Tyr | -0.403* | 0.222 | 0.016 | 0.048 | -0.244 | 0.182 | 0.392 | *-0.063* | *0.088* |
| SDMA | *-0.043* | *0.025* | *-0.627*** | *-0.471*** | *-0.393*** | *0.022* | *0.281* | *0.308* | *-0.262* |
| Trp | 0.314 | 0.405* | 0.450** | 0.481** | 0.665** | 0.403* | -0.141 | *-0.196* | *0.242* |
| Kyn | -0.261 | -0.313 | -0.381* | -0.300 | -0.359* | 0.094 | 0.257 | *0.285* | *-0.359** |
| Cys | *-0.272* | *-0.096* | *-0.345** | *-0.375** | *-0.243* | *0.336** | *0.090* | *-0.130* | *-0.094* |
| Leu/Iso | -0.180 | -0.065 | -0.005 | 0.039 | 0.023 | -0.067 | 0.357 | *0.093* | *0.234* |
| Gln/Glu | 0.057 | 0.183 | -0.006 | -0.025 | 0.006 | 0.303 | -0.196 | *-0.360** | *-0.279* |
| ALB/ Leu | *0.349*** | *-0.011* | *0.423*** | *0.468*** | *0.484*** | *0.179* | *-0.103* | *0.115* | *-0.071* |
| ALB/ Iso | *0.346*** | *0.248* | *0.416*** | *0.619*** | *0.484*** | *0.476*** | *-0.282* | *0.078* | *0.023* |
| ALB /Val | *0.275** | *0.227* | *0.404*** | *0.395*** | *0.531*** | *0.457*** | *-0.215* | *0.033* | *0.155* |
| Pro/Cit | *-0.167* | *0.249* | *0.343** | *0.289* | *0.118* | *0.010* | *-0.069* | *-0.219* | *0.205* |
| Phe/Tyr | 0.291 | -0.207 | -0.044 | -0.061 | 0.062 | -0.076 | 0.147 | *0.041* | *-0.053* |
| Kyn/Trp | -0.288 | -0.453** | -0.445** | 0.380* | -0.506** | -0.128 | 0.079 | *0.345** | *-0.385** |
| Arg/Phe | 0.116 | -0.006 | -0.213 | -0.211 | 0.086 | 0.112 | -0.235 | *-0.226* | *-0.219* |
| SDMA/SCr | *-0.016* | *0.141* | *-0.351*** | *-0.262* | *-0.008* | *0.053* | *-0.166* | *0.004* | *0.399** |
| Ser/Gly | 0.320 | 0.139 | -0.018 | -0.024 | 0.303 | 0.088 | -0.166 | *0.078* | *-0.029* |
| Val/Gly | 0.389* | 0.152 | 0.196 | 0.198 | 0.142 | -0.086 | 0.402 | *-0.211* | *-0.237* |
| Arg/Cit | *0.040* | *0.443*** | *0.007* | *-0.037* | *0.238* | *0.248* | *0.434** | *-0.263* | *-0.172* |
| TAA | -0.334* | -0.169 | -0.114 | -0.114 | -0.194 | 0.224 | 0.015 | *0.078* | *0.355** |
| TEAA | 0.263 | 0.451** | 0.381* | 0.359* | 0.203 | 0.217 | 0.315 | *-0.196* | *0.184* |
| TNEAA | -0.390* | -0.258 | -0.188 | -0.184 | -0.236 | 0.187 | -0.032 | *0.152* | *0.374** |
| TBCAA | 0.350* | 0.375* | 0.516** | 0.473** | 0.068 | -0.048 | 0.280 | *-0.145* | *-0.052* |
| TAAA | -0.105 | 0.296 | 0.183 | 0.236 | 0.093 | 0.408* | 0.345 | *-0.004* | *0.174* |
| TGAA | -0.348* | -0.232 | -0.117 | -0.129 | -0.177 | 0.204 | -0.011 | *0.019* | *0.388** |
| TKAA | 0.090 | 0.474** | 0.301 | 0.290 | 0.194 | 0.335* | 0.201 | *-0.122* | *0.274* |
| TGKAA | -0.070 | 0.326* | 0.242 | 0.254 | 0.147 | 0.392* | 0.222 | *-0.070* | *0.313* |
| TBCAA/Tyr | 0.533** | -0.033 | 0.205 | -0.150 | 0.251 | -0.176 | -0.132 | *-0.070* | *-0.137* |
| ALB/TBAA | *-0.159* | *-0.146* | *-0.246* | *-0.241* | *-0.102* | *0.087* | *0.434** | *0.078* | *0.304* |
| TEAA/TNEAA | 0.423** | 0.371* | 0.214 | 0.239 | 0.348* | 0.115 | 0.195 | *-0.241* | *-0.316* |

* *P*<0.05, ** *P*<0.01.

Italic numbers with underline indicate the Spearman correlation (ρ), and the normal numbers indicate the Pearson correlation (r).

Table S6 Diagnostic performance of biomarkers.

| Marker | AUC (95% CI) | SD | P | Cut off (μg/mL) | sensitivity | specificity |
| --- | --- | --- | --- | --- | --- | --- |
|
| SDMA+Trp | 0.901 (0.860−0.942) | 0.035 | 0.000 | SDMA 0.3240,  Trp 2.0885 | 0.889 | 0.831 |
| SDMA | 0.820 (0.732−0.908) | 0.045 | 0.000 | 0.3240 | 0.833 | 0.763 |
| SDMA/SCr | 0.785 (0.679−0.891) | 0.054 | 0.001 | 2.0885 | 0.917 | 0.697 |
| Trp | 0.738 (0.588−0.889) | 0.077 | 0.005 | 9.4247 | 0.583 | 0.874 |
| Cys | 0.724 (0.608−0.840) | 0.059 | 0.009 | 0.0921 | 0.917 | 0.498 |
| Kyn/Trp | 0.709 (0.616−0.802) | 0.047 | 0.015 | 0.0359 | 0.833 | 0.600 |
| Leu/Iso | 0.661 (0.503−0.818) | 0.080 | 0.061 | 1.8302 | 0.750 | 0.540 |
| Arg | 0.629 (0.483−0.776) | 0.075 | 0.132 | 1.2062 | 0.833 | 0.577 |
| Phe | 0.505 (0.323−0.668) | 0.093 | 0.950 | 12.6370 | 0.500 | 0.312 |
